# CircCDKN2B-AS1 interacts with IMP3 to stabilize hexokinase 2 mRNA and facilitate cervical squamous cell carcinoma aerobic glycolysis progression

**DOI:** 10.1186/s13046-020-01793-7

**Published:** 2020-12-11

**Authors:** Yanan Zhang, Lu Zhao, Shizhou Yang, Yixuan Cen, Tingjia Zhu, Lingfang Wang, Lili Xia, Yuwan Liu, Jian Zou, Junfen Xu, Yang Li, Xiaodong Cheng, Weiguo Lu, Xinyu Wang, Xing Xie

**Affiliations:** 1 Women’s Reproductive Health Laboratory of Zhejiang Province; Women’s Hospital; School of Medicine, Zhejiang University, Hangzhou, 310006 China; 2 Department of Gynecologic Oncology; Women’s Hospital, School of Medicine, Zhejiang University, Hangzhou, No.1 Xueshi Road, Hangzhou, 310006 China

**Keywords:** CircRNA, CircCDKN2B-AS1, Cervical cancer, Aerobic glycolysis, IMP3, HK2

## Abstract

**Background:**

Circular RNAs (circRNAs) have been reported to play key roles in the development of various cancers. However, the biological functions and clinical significance of most circRNAs are still elusive. The purpose of this study was to explore the function and mechanism of a certain circRNA named circCDKN2B-AS1 in cervical cancer development and its potential value in the clinic.

**Methods:**

qRT-PCR was used to verify the expression level of circCDKN2B-AS1. CCK-8, Transwell, and flow cytometry (FCM) assays were performed to detect cellular proliferation, migration, and apoptosis, respectively. A Seahorse XFe96 Analyzer was used to measure glycolysis metabolism level. RNA pull-down, RNA immunoprecipitation (RIP), actinomycin-D addition assays and Western blotting were used to screen and elucidate the potential mechanisms involved. BALB/c nude mice and zebrafish embryos (AB, WT) were used as animal models to investigate tumorigenesis capability. ^18^FDG-microPET/CT imaging and lactic acid (LA) and pyruvic acid (PA) content detection assays were used to detect the level of glucose metabolism in subcutaneous tumors from nude mice.

**Results:**

CircCDKN2B-AS1, a circular isoform of the long noncoding RNA (lncRNA) CDKN2B-AS1, was upregulated in cervical cancer and precancerous tissues. We found that circCDKN2B-AS1 associated with the IMP3 protein depending on a specific binding site and regulated the stability of Hexokinase 2 (HK2) mRNA, the rate-limiting enzyme of the aerobic glycolysis pathway. The expression level of circCDKN2B-AS1 fated the binding of IMP3 to the 3′ untranslated region (UTR) of HK2 mRNA, consequently affecting the malignant cell phenotype and aerobic glycolysis in cervical cancer in vitro and in vivo. Mutant circCDKN2B-AS1, lacking the IMP3 binding site, did not have such effects. Utilization of an inhibitory peptide to block the interaction between circCDKN2B-AS1 and the IMP3 protein impeded the binding of IMP3 to the 3’UTR of HK2 mRNA and suppressed aerobic glycolysis in cervical cancer cells.

**Conclusions:**

Our findings demonstrate that circCDKN2B-AS1 facilitates aerobic glycolysis by sponging the IMP3 protein to stabilize HK2 mRNA, consequently promoting the malignant phenotype in cervical cancer, which may provide a potential approach for cervical cancer therapeutics.

## Background

Cervical cancer is the fourth most common cancer among women globally, leading to an estimated 530,000 new cases and 270,000 deaths each year [1, 2]. Screening and human papillomavirus (HPV) vaccination have remarkably reduced the incidence of cervical cancer in developed countries, but the application of both methods is restricted, and advanced cervical cancer is still very common in low-income countries [3], where approximately 90% of the world’s cervical cancer-related deaths occur [4–6]. The curative effect is not ideal, and the 5-year survival rate is only 16.7% in advanced cervical cancer patients [7]. New therapeutic methods are expected for cervical cancer.

Circular RNAs (circRNAs), formed by a covalently closed loop, are a class of noncoding RNAs [8, 9]. CircRNAs resist RNaseR digestion and are more stable than their linear isoforms [9–11]. Recently, circRNAs have been increasingly recognized as being implicated in gene regulatory networks and influencing diseases [12, 13]. The effect of circRNAs on the development of various cancers, such as breast cancer, oral cancer and hepatocellular carcinoma, has been reported [14–16]. Most of the discoveries have revealed that circRNAs can function by sponging microRNAs and regulating the expression levels of target genes [17, 18]. Recent studies have showed that circRNAs can bind to RNA binding proteins (RBPs) [19–21]; however, the biological function and potential value of the circRNA-RBP complex is still elusive.

IMP3, also known as IGF2BP3, is an RBP. A previous study revealed that IMP3 is highly expressed in various cancers and can regulate the expression of various genes at the posttranscriptional level [22–24]. HK2 is one of the rate-limiting enzymes in the glycolysis pathway and has been recognized to regulate the malignant phenotype of cancer cells (i.e., cellular apoptosis and migration capabilities) [25–27]. Aerobic glycolysis is the energy metabolism characteristic of tumor cells and fates the malignant phenotype of tumor cells, including cervical cancer cells [28–31]. Subcutaneous tumor formation in BALB/c nude mice combined with microPET/CT can be used to effectively explore tumorigenesis and the level of glucose metabolism in tumors [32]. Zebrafish embryo injection is a novel model for cancer research that has the advantages of a short experimental period and a simple operation procedure [33].

In our previous circRNA sequencing analysis in normal and cervical cancer tissues, we identified a circular isoform of the long noncoding RNA (lncRNA) CDKN2B-AS1, named circCDKN2B-AS1. We verified that circCDKN2B-AS1 was upregulated in HPV16-positive cervical cancer and precancerous tissues compared with normal tissues. We further identified that circCDKN2B-AS1 facilitates aerobic glycolysis by interacting with the IMP3 protein to stabilize HK2 mRNA, consequently promoting the malignant phenotype in cervical cancer cells. To better explore the potential therapeutic role of circCDKN2B-AS1, we selected the above two animal models and verified the capability of circCDKN2B-AS1 to regulate HK2 expression and glycolysis by binding IMP3 protein in vivo. Our study may provide a potential approach for cervical cancer therapeutics.

## Methods

### Human tissue samples

In total, 46 normal cervical epithelium tissues, 41 high-grade squamous intraepithelial lesions (HSILs), and 75 cervical cancer tissues were collected for qRT-PCR assays. Cervical cancer samples were obtained from patients who underwent radical hysterectomy, HSIL samples were obtained from patients who underwent colposcopy biopsy, and normal cervical tissues were collected from patients who underwent hysterectomy because of benign gynecological diseases. All the samples were collected from September 2015 to September 2020 at Women’s Hospital, Zhejiang University School of Medicine, China. Patients provided informed consent to obtain samples, and the study was subject to approval by the Hospital Ethical Committee. Patient information for all specimens is shown in Additional file 1: Table S1. Tissue samples were stored in RNA storage solution at 4 °C and stored at − 80 °C until use.

### Identification of HPV type

The HPV type of tissue samples was identified using the 21 HPV GenoArray Diagnostic Kit (Innovation Technologies of Hybribio). With the use of PCR principles to amplify extracted HPV DNA from cervical samples, amplified DNA amplicons were then hybridized with immobilized specific HPV probes on a HybriMem membrane under the patented flow-through hybridization technique. The enzyme immunoassay method was applied for color development to obtain test results. HPV-negative specimens used for RNA sequencing were amplified with the common primer my09/11 PCR of HPV L1 to further determine the HPV-negative status.

### RNA extraction and qRT-PCR

RNA was extracted using TRIzol reagent (Invitrogen, Carlsbad, CA, USA) according to the manufacturer’s instructions. For tissue samples used to validate circRNA expression, the RNA integrity number (RIN) was assessed. qRT-PCR analyses were performed using the PrimeScript RT Reagent Kit and SYBR Premix Ex Taq (TaKaRa, Japan). All the primers are presented in Additional file 2: Table S2.

### Cell line culture

The human cervical cancer cell line SiHa and the human embryonic renal cell line HEK293 were obtained from the American Type Culture Collection (ATCC, USA). The human cervical cancer CaSki cell line was obtained from the Cell Resource Center, Shanghai Institute of Life Sciences, Chinese Academy of Sciences (China), where it was tested and authenticated. It was not cultured continuously for more than 3 months. The SiHa cell line was cultured in DMEM (BI, Israel) supplemented with 10% FBS and maintained at 37 °C in 5% CO_2_, whereas the cervical cancer cell line CaSki and the human embryonic renal cell line HEK293 were cultured in RPMI-1640 (BI, Israel) containing 10% FBS.

### RNA-fluorescence in situ hybridization (FISH) assay

CY3-labeled probes targeting the junction point sequence were used to visualize circCDKN2B-AS1 in situ (details of the probes are shown in Additional file 2: Table S2). SiHa and CaSki cells were plated onto slides and incubated overnight. Triton X-100 (0.5%) was added after washing the slides with PBS 3 times and then incubated at room temperature for 15 min. Then, the cells were fixed with 4% paraformaldehyde for 5 min and incubated with 100% ethanol. After discarding ethanol, the denatured probe was added to immerse the slides. Then, the slides were incubated at 37 °C overnight. Nuclei were counterstained with DAPI-antifade solution, and images were taken on a laser confocal microscope (TCS SP2 AOBS).

### RNaseR resistance assay

RNA samples were incubated with or without 2 U/μg RNaseR at 37 °C for 15 min.

### Northern blot assay

The probe targeting the junction point of circCDKN2B-AS1 was synthesized and labeled with digoxigenin (DIG, details of probes are shown in Additional file 2: Table S2). Fifteen micrograms of total RNA was separated by 1% denaturing formaldehyde gel electrophoresis at 25 V overnight and transferred to Hybond-N + membranes (Amersham, UK, RPN303B). Prehybridization was carried out at 50 °C for 2 h in DIG Easy Hyb solution (Roche). Hybridization was performed at 50 °C overnight. The membrane was washed stringently and blocked in blocking solution for 1 h. Blots were detected by anti-DIG antibody staining and recorded using X-ray films with the chemiluminescence substrate CSPD (Roche).

### Western blot assay

Cellular proteins were extracted using lysis buffer. In total, 20 μg of total protein was separated using 10% SurePAGE (GenScript, USA, M00665) and transferred to PVDF membranes (Bio-Rad, USA, 1620177). The antibodies used were as follows: IMP3 (EMD Millipore), HK2 (Abcam), GAPDH (Diagbio) E-cadherin (CST) and β-Catenin (CST).

### Gene knockdown and overexpression

The human circCDKN2B-AS1 linear sequence was inserted into the plasmid vector pLent-EF1a-circRNA-CMV-RFP-P2A-Puro, and lentiviruses stably overexpressing circCDKN2B-AS1 were constructed (Weizhen, Shandong, China). A nonfluorescent circCDKN2B-AS1 overexpression plasmid was constructed for apoptosis detection using the plasmid vector (Weizhen, Shandong, China). The full-length products and the junction point of the circCDKN2B-AS1-overexpressing products were determined by Sanger sequencing after RT-PCR. Short hairpin RNAs (shRNAs) specific to circCDKN2B-AS1 were inserted into the lentiviral vector GV334 (GeneChem, Shanghai, China). Stable cell lines were obtained by puromycin resistance. The human HK2 overexpression plasmid was purchased from GeneChem. All of the small interfering RNA (siRNA) and primer sequences used in this study are presented in Additional file 2: Table S2.

### Cell viability, migration, invasion and apoptosis assays

In vitro cell viability was detected using Cell Counting Kit-8 assays (Dojindo, Japan). Transwell assays were performed at 48 h after transfection: 1 × 10^4^ cells were resuspended in serum-free Opti-MEM medium, and cell invasion/migration was examined in Transwell cell culture chamber filters coated on the upper side with/without Matrigel (Corning Biocoat) as previously described [34]. The wound healing assays we performed by using culture insert (ibidi, Germany, 80,206) according to the instructions of the manufacturer. The apoptotic rates of SiHa and CaSki cells were determined at 72 h after transfection using an Annexin V-FITC/PI Apoptosis Kit (Mutisiences, China, AP101–100-kit). For cells transfected with circCDKN2B-AS1-overexpressing or empty plasmids, apoptosis was induced by incubation with serum-free medium for 6 h.

### Extracellular acidification rate (ECAR)

Assays were performed using a Seahorse XFe96 analyzer (Seahorse Bioscience, Agilent) according to the manufacturer’s instructions. The ECAR was measured using a Seahorse XF Glycolytic Rate Assay Kit (Seahorse Bioscience, Agilent, 103044-100) and a Seahorse XF Glycolysis Stress Test Kit (Seahorse Bioscience, Agilent, 103020-100). The glycolytic capacities of cells were analyzed with the Seahorse XF Glycolysis Rate/Stress Test Report Generator package. The %PER from glycolysis was calculated by subtracting the acidification from CO_2_ produced by the mitochondria.

### Inhibition of glycolysis in cells

We inhibited the glycolysis level of cervical cancer cells in vitro by adding 2-DG (5 mM, Seahorse Bioscience, Agilent,103044-100).

### Biotin-labeled RNA pull-down assay and mass spectrometry analysis

Biotin-labeled RNA pull-down assays were performed using a Pierce™ Magnetic RNA-Protein Pull-down Kit (Thermo Scientific, USA, 20164). Briefly, cell lysates were prepared using standard lysis buffers (Thermo Scientific, USA). Biotin-labeled DNA probes (details of probes are shown in Additional file 2: Table S2) were incubated with streptavidin magnetic beads for 30 min at room temperature with agitation. Cell lysates were incubated with streptavidin magnetic beads at 4 °C overnight. Magnetic beads were thoroughly washed. The proteins bound to the magnetic bead were separated with SurePAGE (GenScript, USA, M00665), and the region of interest was excised and subjected to mass spectrometry analysis (Lumingbio, Shanghai, China).

### RNA immunoprecipitation (RIP) assay

RIP assays were performed with a Magna RIP™ Quad RNA-Binding Protein Immunoprecipitation Kit (Sigma-Aldrich, USA, 17-704) according to the instructions of the manufacturer.

### Determination of mRNA half-life

To assess the half-life of HK2 mRNA, actinomycin-D (5 μg/ml, Sigma, A4262) was added to block mRNA synthesis. Total RNA was collected at different time points and subjected to RT-qPCR analysis. The expression level of HK2 mRNA was normalized to that of 18S rRNA and plotted as a percentage of the value at time 0 (set at 100%).

### Design and synthesis of inhibitory peptides

The inhibitory peptides for blocking the interaction between circCDKN2B-AS1 and IMP3 were designed and synthesized by ChinaPeptides Co. Ltd. (Shanghai, China). The inhibitory peptides were synthesized by linking the biotin-labeled 11-amino acid cell-penetrating peptide YGRKKRRQRRR of the Tat protein transduction domain with the core amino acids of IMP3 at the N-terminus (purity greater than 95%).

### Biotin peptide pull-down assay

Biotin-labeled peptides were incubated with streptavidin magnetic beads (Thermo Scientific, USA, 88817) for 4 h at 4 °C. Then, the bead-peptide complex was incubated with total RNA at 4 °C overnight. Beads were extensively washed, and RNAs that precipitated were measured by qRT-PCR.

### Nude mice xenograft experiments

This study was conducted in full accordance with the ARRIVE guidelines for reporting animal research [35]. All animal experiments were approved by the Animal Ethical and Welfare Committee of Zhejiang Chinese Medical University under an Affidavit of Approval of Animal Ethical and Welfare license (No. IACUC-20190128-01) and in accordance with the Animals (Scientific Procedures) Act, 1986 (UK) (amended 2013). BALB/c nude mice (4 weeks old±1 week, weighing 18 ± 5 g, female) were ordered from Shanghai SLAC Laboratory Animal Company, Ltd. (China). Nude mice were randomly divided into two groups and anesthetized by inhalation of isoflurane gas (2–3%) also under the effect of muscle relaxation. In total, 10^7^ stable SiHa cells were resuspended in 100 μL PBS and injected subcutaneously under the left armpit of 4-week-old nude mice (*n* = 10 per group). The length and width of the subcutaneous tumor were measured once a week for 5 weeks, and the volume of the subcutaneous tumor was calculated according to the following formula: volume (cm^3^) = (length×width^2^)/2. Five weeks after injection, 6 mice in each group were randomly selected and sacrificed by intravenous injection of pentobarbital (100 mg/kg), and subcutaneous tumors were harvested. A portion of the tumor was stored in RNA storage solution to extract RNA, and the remainder of the tumor was fixed with paraformaldehyde for immunohistochemistry and hematoxylin and eosin (HE) staining. The other 4 mice in each group were subjected to microPET/CT scans. Another 20 nude mice (4 weeks old) were randomly divided into 4 groups (*n* = 5 per group) and injected subcutaneously under the left armpit with 10^7^ stable SiHa cells. siRNAs targeting a negative control (NC) or HK2 were mixed with FECT and Opti-MEM medium and injected into the subcutaneous tumor every week. The volume of the subcutaneous tumor was calculated according to the following formula: volume (cm^3^) = (length×width^2^)/2. Four weeks after the injection of cells, mice were sacrificed, and subcutaneous tumors were harvested, weighed, and stored at − 80 °C for RNA extraction, lactic acid (LA)/pyruvic acid (PA) detection and RIP assays.

### MicroPET/CT scans in nude mice

MicroPET/CT imaging of nude mice was performed using the SuperArgus PET/CT 4R system (Sedecal, Spain) 5 weeks after cell injection. Briefly, 4 tumor-bearing nude mice in each group were anesthetized by inhalation of isoflurane gas (2–3%). Then, nude mice were injected with 7.4 MBq (200 μCi) of ^18^F-FDG via the abdominal cavity and scanned at 120 min after injection. Nude mice were subjected to a 10-min microCT scan and then to a 15-min microPET scan. Scans were performed under isoflurane (1–1.5%) inhalation to maintain anesthesia. Images of nude mice were reconstructed manually. The PET/CT camera Vista explore was used to calculate the average standardized uptake volume (SUV_AVG_). Then, the mice were sacrificed by intravenous injection of pentobarbital (100 mg/kg).

### Digestion of subcutaneous tumors

Subcutaneous tumors were digested with 10 mg type 1 collagenase (Worthington, 49E19342, USA) at 37 °C for 60 min. The digestion products were centrifuged at 1200 r for 5 min, and the precipitate was subjected further to RIP assays and RNA extraction.

### Detection of LA and PA

The levels of LA and PA in 10 mg subcutaneous tumors from each mouse were detected by using an LA detection kit (Solarbio, China, BC2230) and a PA detection kit (Solarbio, China, BC2200).

### Zebrafish embryo model

Zebrafish (AB, wild type [WT], 48-h embryo) embryos were obtained from the Core Facilities, Zhejiang University, School of Medicine, cultured in Petri dishes with an appropriate amount of water and placed in a 28 °C incubator under constant temperature and light. Then, 0.003% 1-phenyl-2-thiourea (PTU) (Sigma) was added within 24 h after the embryo was produced. In total, 200 SiHa cells were harvested after transfection and resuspended in PBS. Plasma membranes were stained with red-fluorescent Alexa Fluor® 594 wheat germ agglutinin of the Image-iT™ LIVE Plasma Membrane and Nuclear Labeling Kit (Molecular Probes, USA, I34406). Zebrafish embryos were anesthetized with 0.016% tricaine (Sigma). Then, the cells were injected into the yolk sac of zebrafish embryos. Then, we obtained images with a fluorescence microscope at 48 h after injection. The area of red fluorescence in the zebrafish yolk sac was calculated using ImageJ software [33]. Next, the zebrafish embryos were anesthetized with an overdose of tricaine.

### Immunohistochemical staining

Immunohistochemical staining with an antibody specific to HK2 (Proteintech, USA) and quantitative evaluation were performed as previously described [36].

### Statistical analysis

Statistical significance was calculated by using GraphPad Prism 5 software. All experiments were repeated at least three times.

## Results

### CircCDKN2B-AS1 is upregulated in cervical cancer and precancerous tissues

**Fig. 1:**
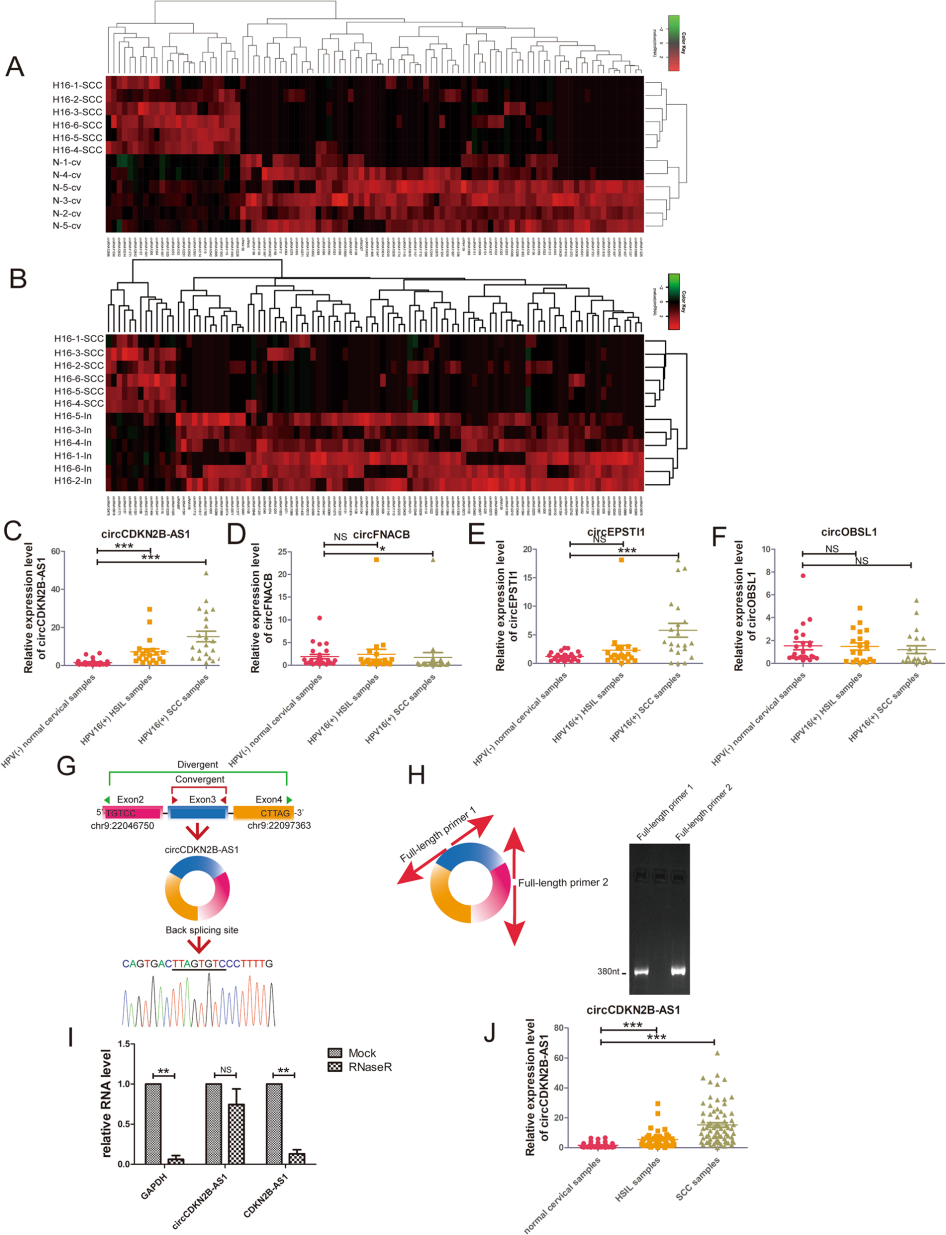
CircCDKN2B-AS1 is upregulated in cervical cancer and precancerous tissues. **a** Heatmap of the top 100 differentially expressed circRNAs between HPV16-positive cervical squamous cell carcinoma and HPV-negative normal cervical epithelium tissues. **b** Heatmap of the top 100 differentially expressed circRNAs between HPV16-positive cervical squamous cell carcinoma and HPV16-positive normal cervical epithelium tissues. **c** Expression levels of circCDKN2B-AS1 in 25 HPV-negative cervical normal epithelium samples, 20 HPV 16-positive HSIL samples and 21 HPV 16-positive squamous cell carcinoma samples were detected with junction point-specific primers (mean ± SEM, Kruskal-Wallis test). **d** Expression levels of circFNACB in 25 HPV-negative cervical normal epithelium samples, 20 HPV 16-positive HSIL samples and 21 HPV 16-positive squamous cell carcinoma samples were detected with junction point-specific primers (mean ± SEM, Kruskal-Wallis test). **e** Expression levels of circEPSTI1 in 25 HPV-negative cervical normal epithelium samples, 20 HPV 16-positive HSIL samples and 21 HPV 16-positive squamous cell carcinoma samples were detected with junction point-specific primers (mean ± SEM, Kruskal-Wallis test). **f** Expression levels of circOBSL1 in 25 HPV-negative cervical normal epithelium samples, 20 HPV 16-positive HSIL samples and 21 HPV16-positive squamous cell carcinoma samples were detected with junction point-specific primers (mean ± SEM, Kruskal-Wallis test). **g** Upper panel: the location of CDKN2B-AS1 in the genomic region and the circularization of exon 2, exon 3 and exon 4 of CDKN2B-AS1 transcript variant 4. Convergent (red) and divergent (green) primers were designed to amplify the linear or backsplicing products. Lower panel: sequencing analysis of the backsplicing products showing the junction in circCDKN2B-AS1. **h** Left: schematic diagram of the full-length primers. Right: full-length circCDKN2B-AS1 was amplified with two pairs of full-length primers designed to target two different points of circCDKN2B-AS1. **i** qRT-PCR assays were performed to determine GAPDH, circCDKN2B-AS1, and linear CDKN2B-AS1 levels in RNA samples of SiHa cells with and without RNaseR treatment (mean ± SEM, *n* = 3; unpaired Student’s t-test). **j** Expression levels of circCDKN2B-AS1 in 46 cervical normal epithelium samples, 41 HPV-positive HSIL samples and 75 HPV-positive squamous cell carcinoma samples were detected with junction point-specific primers (mean ± SEM, Kruskal-Wallis test). *P < 0.05, **P < 0.01, ***P < 0.001

To explore circRNA role in cervical cancer development, we analyzed aberrantly expressed circRNAs among 6 HPV16 positive cervical squamous-cell carcinoma, 6 HPV16 positive normal cervical epithelium, and 6 HPV negative normal cervical epithelium tissues in our circRNA sequencing data, available in the Gene Expression Omnibus (GEO) database (GSE147009) (Fig. 1a-b). We selected 4 circRNAs (circCDKN2B-AS1, circFNACB, circEPSTI1, and circOBSL1) that were upregulated or downregulated in HPV16 positive cervical squamous-cell carcinoma compared with both HPV16 positive and negative normal cervical epithelium samples for further validation.

The expression levels of these 4 circRNAs were detected in 25 HPV-negative normal cervical tissues, 20 HPV16-positive HSIL, and 21 HPV16-positive cervical cancer by qRT-PCR. Among these circRNAs, a novel circRNA formed by the circularization of exon 2, exon 3 and exon 4 of CDKN2B-AS1 was significantly aberrantly expressed in both HSIL tissues and cancer tissues compared with normal cervical tissues (Fig. 1c-f). Products amplified by divergent primers of these four circRNAs were confirmed by Sanger sequencing (Fig. 1g, Additional file 3: Fig. S1A-C, Additional file 4, Table S3).

CircCDKN2B-AS1 was formed by the circularization of exon 2, exon 3 and exon 4 of CDKN2B-AS1 (Fig. 1g). Linear CDKN2B-AS1 is a lncRNA that has been reported to facilitate tumorigenesis in several cancers, such as breast cancer and gastric cancer. To verify the endogenous circularization of exons 2–4 of CDKN2B-AS1, we designed convergent primers and divergent primers to amplify the linear and circular forms of CDKN2B-AS1. PCR using the convergent primers amplified products in both genomic DNA and cDNA formed by reverse transcription, but the divergent primers only amplified products in cDNA (Additional file 3: Fig. S1D). Products amplified by divergent and convergent primers were confirmed by Sanger sequencing (Additional file 4, Table S3). RT-PCR products amplified by two pairs of full-length primers designed in two different points of circCDKN2B-AS1 were used to amplify full-length circCDKN2B-AS1 (Fig. 1h), and the sequence of the products was confirmed by Sanger sequencing (Additional file 4, Table S3). CircCDKN2B-AS1 was more resistant to RNaseR digestion than the canonical form of CDKN2B-AS1 (Fig. 1i).

We noted that backsplicing of the novel 380-nt circCDKN2B-AS1 also formed a 1719 nt isoform, as reported in circBase (hsa_circ_0008796). However, the length of this circCDKN2B-AS1 annotated in CircPrimer software was 380 nt instead of 1719 nt [37]. A previous study reported the alternative splicing of circRNAs and confirmed the length of circRNAs using Northern blot analysis [38]. Northern blotting results showed that the length of the main isoform of circCDKN2B-AS1 in cervical cancer cell lines was 380 nt (Additional file 3: Fig. S1E).

In addition, we detected the level of circCDKN2B-AS1 expression by qRT-PCR assay in 46 normal cervical tissues, 41 HSIL, and 75 cervical cancer tissues regardless HPV positive or negative, and found that circCDKN2B-AS1 expression was upregulated in HSIL and cervical cancer, and the level of circCDKN2B-AS1 expression in cervical cancer and HSIL tissues was 15.16 and 5.48-fold, respectively, compared to normal cervical tissues (Fig. 1j). Then we analyzed the correlation between the level of circCDKN2B-AS1 expression and clinical prognostic parameters in 75 cervical cancer patients, and found that the level of circCDKN2B-AS1 expression was positively correlated with poorer prognostic parameters including tumor size, FIGO stage, deep stromal invasion, and lymph node metastasis (Table 1).

**Table 1:** Association between circCDKN2B-AS1 expression level and clinicopathological parameters of cervical squamous carcinoma patients. (Bold values were presented as P 0.05)

	circCDKN2B-AS1 expression		
	High	Low	***P*** value
**Age (years)**			0.203
**< 35**	6	4	
**≥ 35**	51	14	
**FIGO stage**			**0.022**
I**B**	34	16	
II**A**	23	2	
**Tumor size** (**cm**)			**0.029**
**< 4**	35	16	
**≥ 4 cm**	22	2	
**Deep stromal invasion**			
**< 66%**	20	13	**0.006**
**≥ 66%**	37	5	
**Lymph nodes Metastasis**			**0.044**
**negative**	46	18	
**positive**	11	0	

### CircCDKN2B-AS1 facilitates the malignant phenotype of cervical cancer cells

**Fig. 2:**
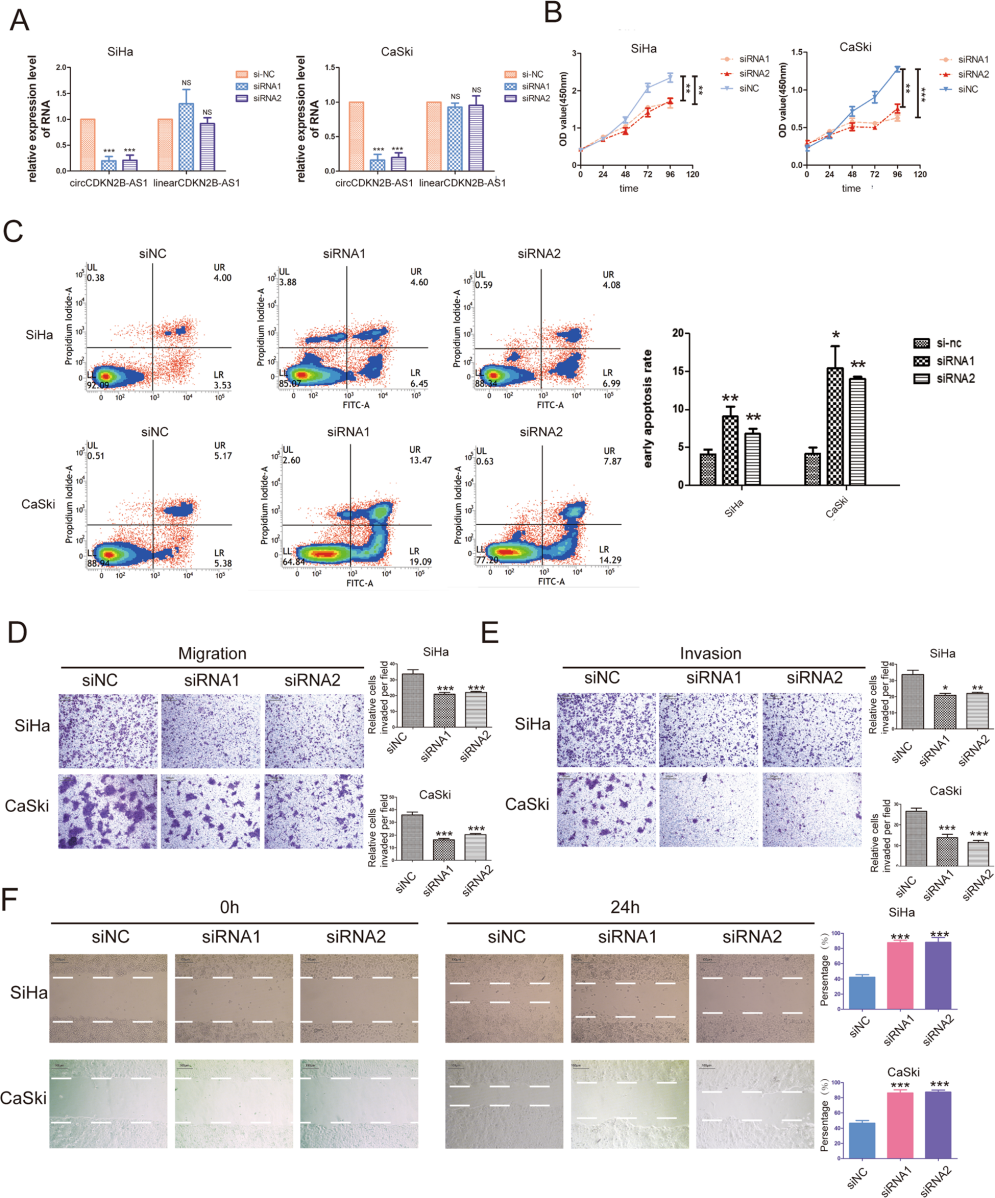
CircCDKN2B-AS1 knockdown suppresses the growth and vitality of cervical cancer cells. **a** The levels of circCDKN2B-AS1 and linear CDKN2B-AS1 expression in SiHa (left panel) and CaSki (right panel) cells after transfection with two circCDKN2B-AS1 backsplicing-specific siRNAs or a negative control siRNA were determined by qRT-PCR (mean ± SEM, n = 3, one-way ANOVA). **b** The growth curve was determined with CCK-8 assays after transfection with two circCDKN2B-AS1 backsplicing-specific siRNAs or a negative control siRNA. Left panel, SiHa cells; right panel, CaSki cells (mean ± SEM, *n* = 3, one-way ANOVA). **c** Apoptosis level after transfection with two circCDKN2B-AS1 backsplicing-specific siRNAs or a negative control siRNA. Left upper panel, SiHa cells; left lower panel, CaSki cells; right panel, the quantification results of early apoptosis (mean ± SEM, n_SiHa_ = 4, n_CaSki_ = 3, one-way ANOVA). **d** Left panel: representative images of the migration capability of SiHa and CaSki cells transfected with two circCDKN2B-AS1 backsplicing-specific siRNAs or a negative control siRNA. Right panel: quantification of migration assays (mean ± SEM, *n* = 9, one-way ANOVA). **e** Left panel: representative images of the invasion capability of SiHa and CaSki cells transfected with two circCDKN2B-AS1 backsplicing-specific siRNAs or a negative control siRNA. Right panel: quantification of invasion assays (mean ± SEM, n = 9, one-way ANOVA). **f** Left panel: representative images of wound healing assays of SiHa and CaSki cells transfected with two circCDKN2B-AS1 backsplicing-specific siRNAs or a negative control siRNA. Right panel: quantification of wound healing assays (mean ± SEM, *n* = 3, one-way ANOVA). *P < 0.05, **P < 0.01, ***P < 0.001

To investigate the biological function of circCDKN2B-AS1 in the malignant phenotype of cervical cancer cells, we knocked down circCDKN2B-AS1 by using two specific siRNAs in SiHa and CaSki cells and found that circCDKN2B-AS1 downregulation did not affect the expression of the linear forms of CDKN2B-AS1 (Fig. 2a) but inhibited cellular proliferation, migration and invasion, and promoted cellular apoptosis (Fig. 2b-f,Additional file 5: FigureS2).

We next observed the role of circCDKN2B-AS1 overexpression in SiHa and CaSki cells using a constructed plasmid to upregulate circCDKN2B-AS1 expression in SiHa and CaSki cells (Additional file 6: Fig. S3A). Sanger sequencing followed by RT-PCR verified that the sequence of circCDKN2B-AS1 was correct (Additional file 4, Table S3). We found that circCDKN2B-AS1 overexpression promoted cellular proliferation, migration and invasion and repressed the cellular apoptotic rate (Additional file 6: Fig. S3B-E). Collectively, these data are consistent with the notion that circCDKN2B-AS1 facilitates the malignant phenotype of cervical cancer and acts as a tumor promoter in cervical cancer development.

### CircCDKN2B-AS1 cooperates with IMP3 to promote glycolysis in cervical cancer

**Fig. 3:**
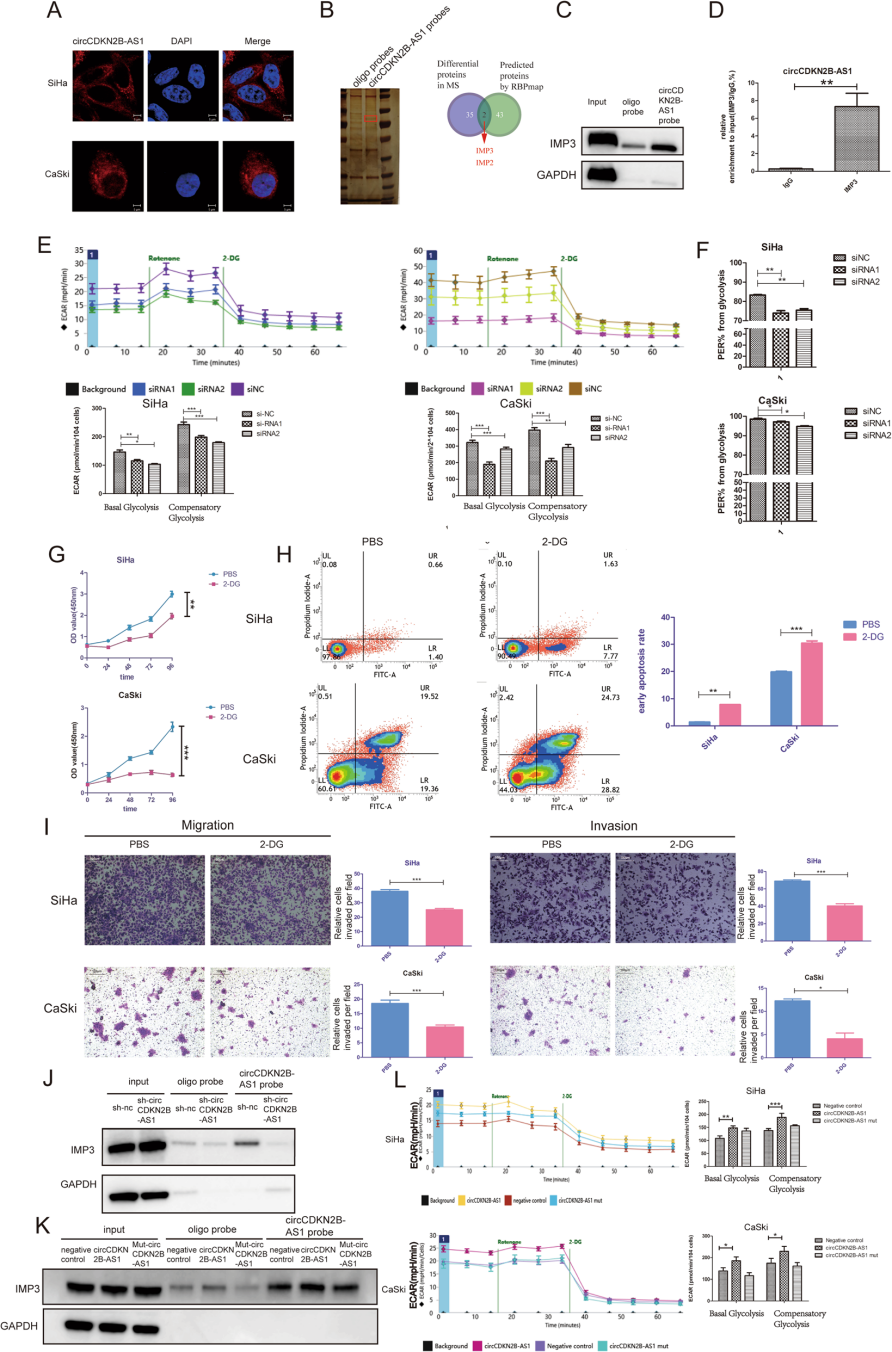
CircCDKN2B-AS1 cooperates with IMP3 to promote glycolysis in cervical cancer. **a** The localization of circCDKN2B-AS1 in cytoplasm in SiHa and CaSki cells by RNA-FISH assays. CircCDKN2B-AS1 was labeled with a specific probe (red) and the nucleus was stained with DAPI (blue). **b** Left: differential proteins pulled down by the circCDKN2B-AS1 or the oligo probe in SiHa cells. The red box indicating the differential proteins; Right: their overlap with those proteins obtained from the RBPmap analysis. **c** Western blot after RNA pull-down assays showing the IMP3 protein pulled down by biotin-labeled circCDKN2B-AS1 probes from the lysates of SiHa cells. **d** qRT-PCR after RIP assays showing circCDKN2B-AS1 recruited by the IMP3 protein from the lysates of SiHa cells (mean ± SEM, *n* = 3, unpaired Student’s t-test). **e** Upper: the ECAR in SiHa and CaSki cells transfected with two siRNAs or a negative control siRNA with Seahorse XFe assays. Lower: quantification of basal glycolysis and compensatory glycolysis in two cells (mean ± SEM, *n* = 6, one-way ANOVA). **f** Quantification of the %PER from the glycolysis of SiHa and CaSki cells transfected with two siRNAs or a negative control siRNA (mean ± SEM, n = 6, unpaired Student’s t-test). **g**-**i** The growth curve (**g**), apoptosis level (**h**), migration and invasion (**i**) of SiHa and CaSki cells after addition of 2-DG or PBS (mean ± SEM, n = 3, unpaired Student’s t-test). **j** Western blot assay showing the level of IMP3 protein pulled down by biotin-labeled circCDKN2B-AS1 probes from lysates of SiHa cells with or without stable circCDKN2B-AS1 knockdown. **k** Western blot assay showing the level of IMP3 protein pulled down by biotin-labeled circCDKN2B-AS1 probes from the lysates of HEK293 cells following transfection with the WT/mutant circCDKN2B-AS1 overexpression vector or the negative control vector. **l** Left: the ECAR in SiHa and CaSki cells transfected with the negative control plasmid, the circCDKN2B-AS1-overexpressing plasmid, and the mutant circCDKN2B-AS1-overexpressing plasmid with Seahorse XFe assays. Right: quantification of basal glycolysis and compensatory glycolysis in SiHa and CaSki cells (mean ± SEM, *n* = 6, unpaired Student’s t-test). *P < 0.05, **P < 0.01, ***P < 0.001

RNA-FISH showed that circCDKN2B-AS1 was mainly localized in the cytoplasm of SiHa and CaSki cells (Fig. 3a), implying that circCDKN2B-AS1 probably regulated gene expression at the posttranscriptional level by sponging microRNAs or RBPs.

Then, we performed RNA pull-down assays to determine the RBPs that interact with circCDKN2B-AS1. Mass spectrometry analysis after RNA pull-down assays revealed 35 differential proteins between the oligo probe and the circCDKN2B-AS1-specific probe (Fig. 3b, Additional file 7: Fig. S4A-B, Additional file 8: Table S4). Then, we utilized the RBPmap database to predict candidate RBPs that interact with circCDKN2B-AS1. The results showed 43 proteins that may interact with circCDKN2B-AS1 (Additional file 9: Table S5). Of these, IMP2 and IMP3 were included in both the mass spectrometry analysis and the RBPmap database (Fig. 3b). Moreover, IMP3 showed higher coverage [%] in the mass spectrometry results (Additional file 8: Table S4) and higher Z-scores in the RBPmap results (Additional file 9: Table S5). Thus, IMP3 protein was more possibly to be interacted with circCDKN2B-AS1. RIP and RNA pull-down assays confirmed the interaction between circCDKN2B-AS1 and IMP3 in SiHa and CaSki cells (Fig. 3c-d, Additional file 7: Fig. S4C). These data suggest that circCDKN2B-AS1 interacts with the IMP3 protein in cervical cancer cells.

A previous study showed that the IMP3 protein was a protein partner of RNAs or a protein that regulates glycolysis levels in cancer cells [39, 40]. Thus, we investigated the role of circCDKN2B-AS1 in modulating glycolysis in cervical cancer cells. We found that the ECAR and %PER from glycolysis were decreased with circCDKN2B-AS1 knockdown in SiHa and CaSki cells (Fig. 3e-f, Additional file 7: Fig. S4D), suggesting that circCDKN2B-AS1 knockdown weakens glycolysis in cervical cancer cells. We further used 2-DG to inhibit glycolysis in cervical cancer cells, and found that the inhibition of glycolysis decreased cellular proliferation, migration and invasion, and increased apoptotic rate in cervical cancer cells (Fig. 3g-i).

We also found that IMP3 protein expression was not affected by circCDKN2B-AS1 knockdown in SiHa and CaSki cells; however, the amount of IMP3 protein recruited by circCDKN2B-AS1 was decreased with circCDKN2B-AS1 knockdown (Fig. 3j).

Additionally, we designed a mutant circCDKN2B-AS1 overexpression vector with the mutation in the binding site (AAAAACACACA) predicted by RBPmap. We verified the interaction between circCDKN2B-AS1 and IMP3 through the specific binding site in HEK293 cells, and we found that IMP3 was recruited by upregulated WT circCDKN2B-AS1 but not by upregulated mutant circCDKN2B-AS1 in HEK293 cells (Fig. 3k). Consistent with these results, WT circCDKN2B-AS1 overexpression promoted glycolysis in SiHa and CaSki cells, but this phenomenon was reversed by mutating the binding site between circCDKN2B-AS1 and IMP3 (Fig. 3l, Additional file 7: Fig. S4E-F). Together, our results suggest that circCDKN2B-AS1 cooperates with IMP3 to promote glycolysis of cervical cancer cells.

### CircCDKN2B-AS1 regulates the stability of HK2 mRNA via recruiting IMP3 protein

**Fig. 4:**
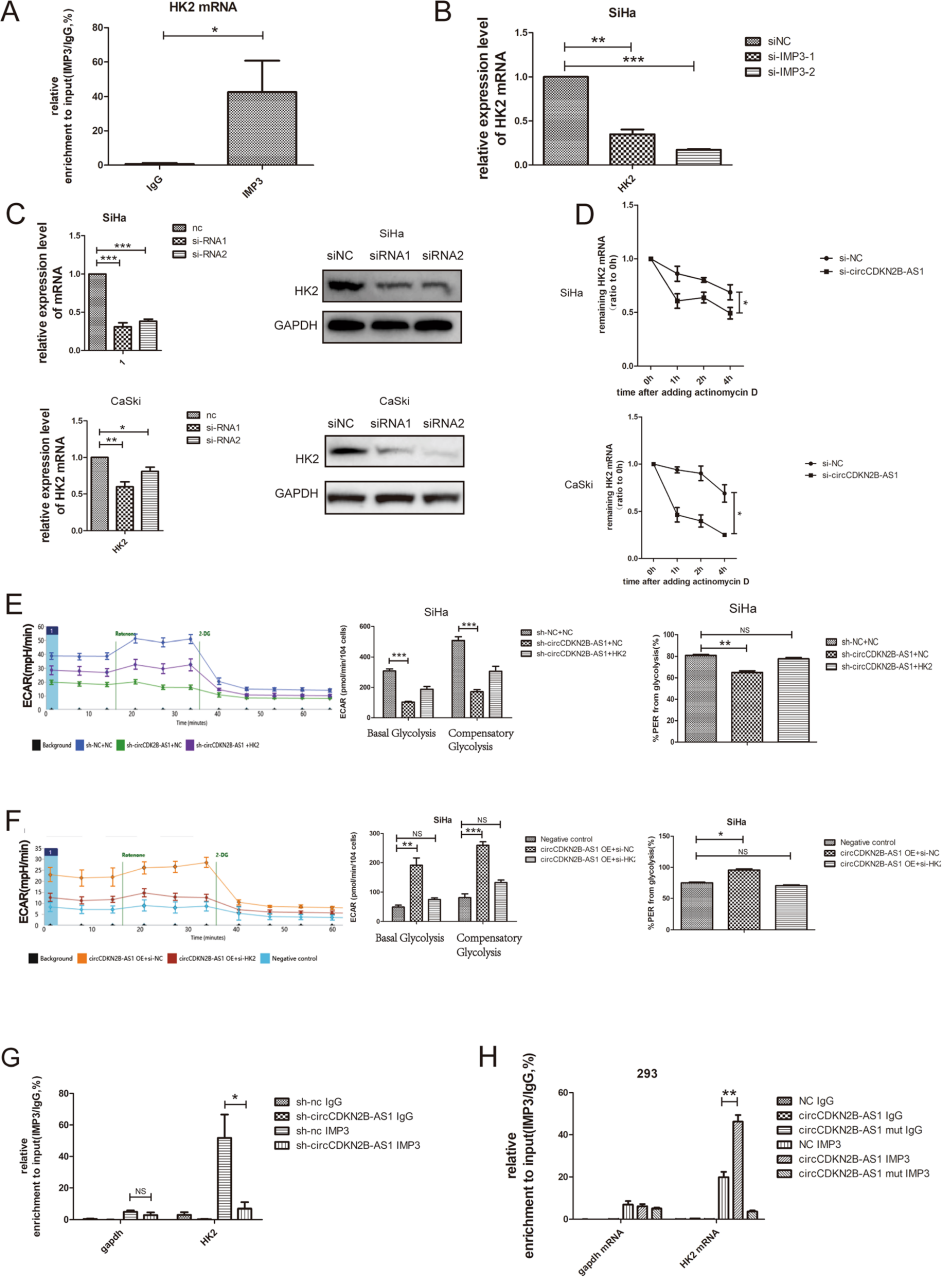
CircCDKN2B-AS1 regulates the stability of HK2 mRNA by recruiting IMP3. **a** qRT-PCR after RIP assays showing the 3’UTR of HK2 mRNA recruited by the IMP3 protein from the lysates of SiHa cells (mean ± SEM, n = 3, unpaired Student’s t-test). **b** The levels of HK2 mRNA expression following IMP3 knockdown using two siRNAs or a negative control siRNA in SiHa cells (mean ± SEM, *n* = 5, one-way ANOVA). **c** Left: the levels of HK2 mRNA expression after transfection with two circCDKN2B-AS1 siRNAs or a negative control siRNA in SiHa and CaSki cells (mean ± SEM, n_SiHa_ = 5, n_CaSki_ = 3, one-way ANOVA). Right: the levels of HK2 protein expression in SiHa and CaSki cells. **d** Actinomycin D was added to block RNA synthesis. The levels of HK2 mRNA in SiHa and CaSki cells with circCDKN2B-AS1 knockdown or not (mean ± SEM, *n* = 4, unpaired Student’s t-test). **e** Left: the ECAR in SiHa cells with stably transfected sh-circCDKN2B-AS1, or a negative control shRNA, or sh-circCDKN2B-AS1 plus HK2 overexpression plasmids, using Seahorse XFe assays. Middle: basal glycolysis and compensatory glycolysis. Right: Quantification of the %PER. (mean ± SEM, n = 6, one-way ANOVA). **f** Left: the ECAR in SiHa cells with lentivirus stably overexpressing circCDKN2B-AS1, or a negative control lentivirus, or lentivirus stably overexpressing circCDKN2B-AS1 plus HK2 siRNA, using Seahorse XFe assays. Middle: basal glycolysis and compensatory glycolysis. Right: Quantification of the %PER. (mean ± SEM, n = 3, one-way ANOVA). **g** qRT-PCR following RIP assays showing the 3’UTR of HK2 mRNA recruited by the IMP3 protein from the lysates of SiHa cells with or without stable circCDKN2B-AS1 knockdown (mean ± SEM, n = 3, unpaired Student’s t-test). **h** qRT-PCR following RIP assays showing the 3’UTR of HK2 mRNA recruited by the IMP3 protein from the lysates of HEK293 cells following transfection with the WT/mutant circCDKN2B-AS1 overexpression or the negative control vector (mean ± SEM, n = 3, unpaired Student’s t-test). *P < 0.05, **P < 0.01, ***P < 0.001

Then, we compared the differentially expressed mRNAs obtained with our RNAseq analysis (available in GSE147010) and the CLIP-Seq analysis of IMP3 in the POSTAR2 database (http://lulab.life.tsinghua.edu.cn/postar/) and found that HK2, ENO1, PGK1 and PKM were the potential downstream molecules of circCDKN2B-AS1 and IMP3. Among these molecules, HK2 mRNA was the most significantly downregulated in SiHa cells following circCDKN2B-AS1 knockdown (Additional file 10: Fig. S5A). While a previous study showed that the IMP3 protein could be recruited to stabilize HK2 mRNA [38]. We thus suspected that HK2 was the common downstream molecule of circCDKN2B-AS1 and IMP3. We found that the 3′ untranslated region (UTR) of HK2 mRNA was significantly recruited by the IMP3 protein (Fig. 4a). Accordingly, knockdown of IMP3 reduced the expression of HK2 mRNA in SiHa cells (Fig. 4b). We further observed the significantly downregulated expression of both HK2 mRNA and protein in SiHa and CaSki cells following circCDKN2B-AS1 knockdown (Fig. 4c) and that the stability of HK2 mRNA was lower in SiHa and CaSki cells following circCDKN2B-AS1 knockdown than in control cells (Fig. 4d).

Furthermore, we constructed a SiHa cell line with stable circCDKN2B-AS1 knockdown using shRNAs and with circCDKN2B-AS1 overexpression using lentivirus, respectively. We found that cells with stable circCDKN2B-AS1 knockdown exhibited suppressed glycolysis compared to control cells, but this suppression of glycolysis was reversed by HK2 overexpression (Fig. 4e, Additional file 10: Fig. S5B), vice versa (Fig. 4f).

In addition, the percentage of HK2 mRNA 3’UTR recruited to IMP3 were decreased after circCDKN2B-AS1 knockdown in SiHa cells (Fig. 4i) and increased after circCDKN2B-AS1 overexpression in HEK293 cells, but mutant circCDKN2B-AS1 didn’t have such effects (Fig. 4j). Taken together, these results suggest that circCDKN2B-AS1 cooperates with IMP3 to regulate the stability of HK2 mRNA.

### Inhibitory peptides repress glycolysis by blocking the interaction between the circCDKN2B-AS1 and IMP3 protein in cervical cancer cells

**Fig. 5:**
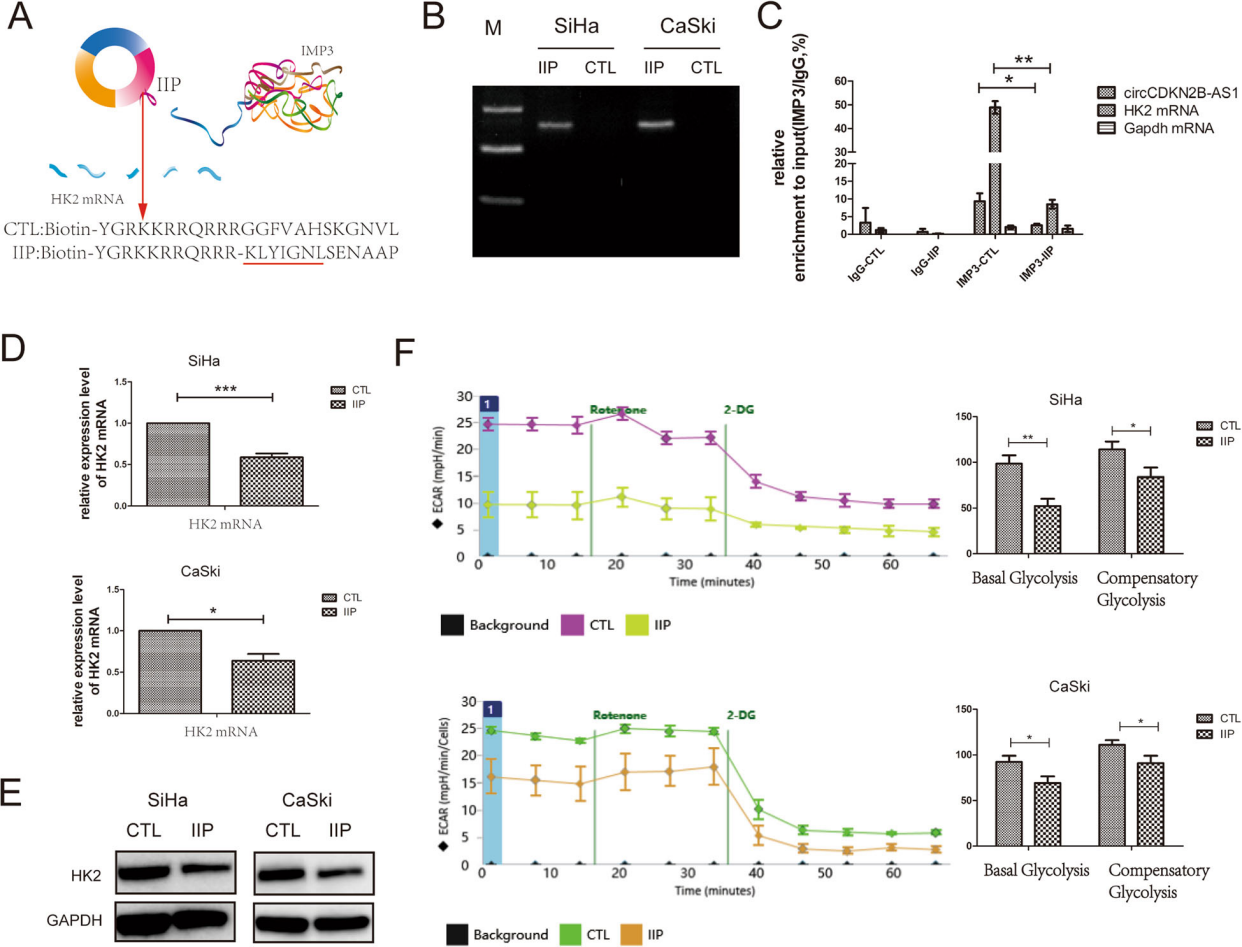
Inhibitory peptides repress glycolysis by blocking the interaction between the IMP3 protein and circCDKN2B-AS1 in cervical cancer cells. **a** Upper: schematic illustration showing the structure and core amino acids of the IMP3 protein that binds to circCDKN2B-AS1. Lower: amino acid sequence of the inhibitory peptide containing the core amino acids of IMP3 (IIP) and the control inhibitory peptide (CTL). **b** Biotin-labeled peptide pull-down assays showing the interaction of IIP or CTL with circCDKN2B-AS1 in SiHa and CaSki cells. **c** qRT-PCR following RIP assays showing circCDKN2B-AS1 and the 3’UTR of HK2 mRNA recruited by the IMP3 protein from the lysates of SiHa cells with the addition of IIP or CTL (mean ± SEM, n = 3, unpaired Student’s t-test). **d** HK2 mRNA expression levels in SiHa and CaSki cells with the addition of IIP or CTL, using qRT-PCR (mean ± SEM, n = 3, unpaired Student’s t-test). **e** HK2 protein expression levels in SiHa and CaSki cells with the addition of IIP or CTL, using Western blot. **f** Left: the ECAR in SiHa and CaSki cells with the addition of IIP or CTL using Seahorse XFe assays. Right: quantification of basal glycolysis and compensatory glycolysis (mean ± SEM, n = 6, unpaired Student’s t-test). *P < 0.05, **P < 0.01, ***P < 0.001

It has been reported that inhibitory peptides can block the interaction between specific circRNAs and RBPs [41, 42]. We used an inhibitory peptide to block the binding between circCDKN2B-AS1 and IMP3. Based on the catRapid and RNABindRPlus prediction results, we designed cell-penetrating inhibitory peptides that include the core amino acids of IMP3 and RRM1, named IIP (Fig. 5a), and confirmed that IIP could interact with circCDKN2B-AS1 in SiHa and CaSki cells (Fig. 5b). Accordingly, we found that the interaction between circCDKN2B-AS1 and IMP3 became weaker after SiHa and CaSki cells were treated with IIP (Fig. 5c). Consequently, the binding between IMP3 and the 3’UTR of HK2 mRNA (Fig. 5c) and HK2 expression were reduced (Fig. 5d-e). Then, we detected the glycolysis capabilities of IIP-treated cells and found that glycolysis in SiHa and CaSki cells treated with IIP was also decreased (Fig. 5f), suggesting that IIP represses glycolysis by blocking the interaction between circCDKN2B-AS1 and the IMP3 protein in cervical cancer cells.

### CircCDKN2B-AS1 facilitates aerobic glycolysis and cancer progression via interacting with IMP3 and HK2 mRNA in vivo

**Fig. 6:**
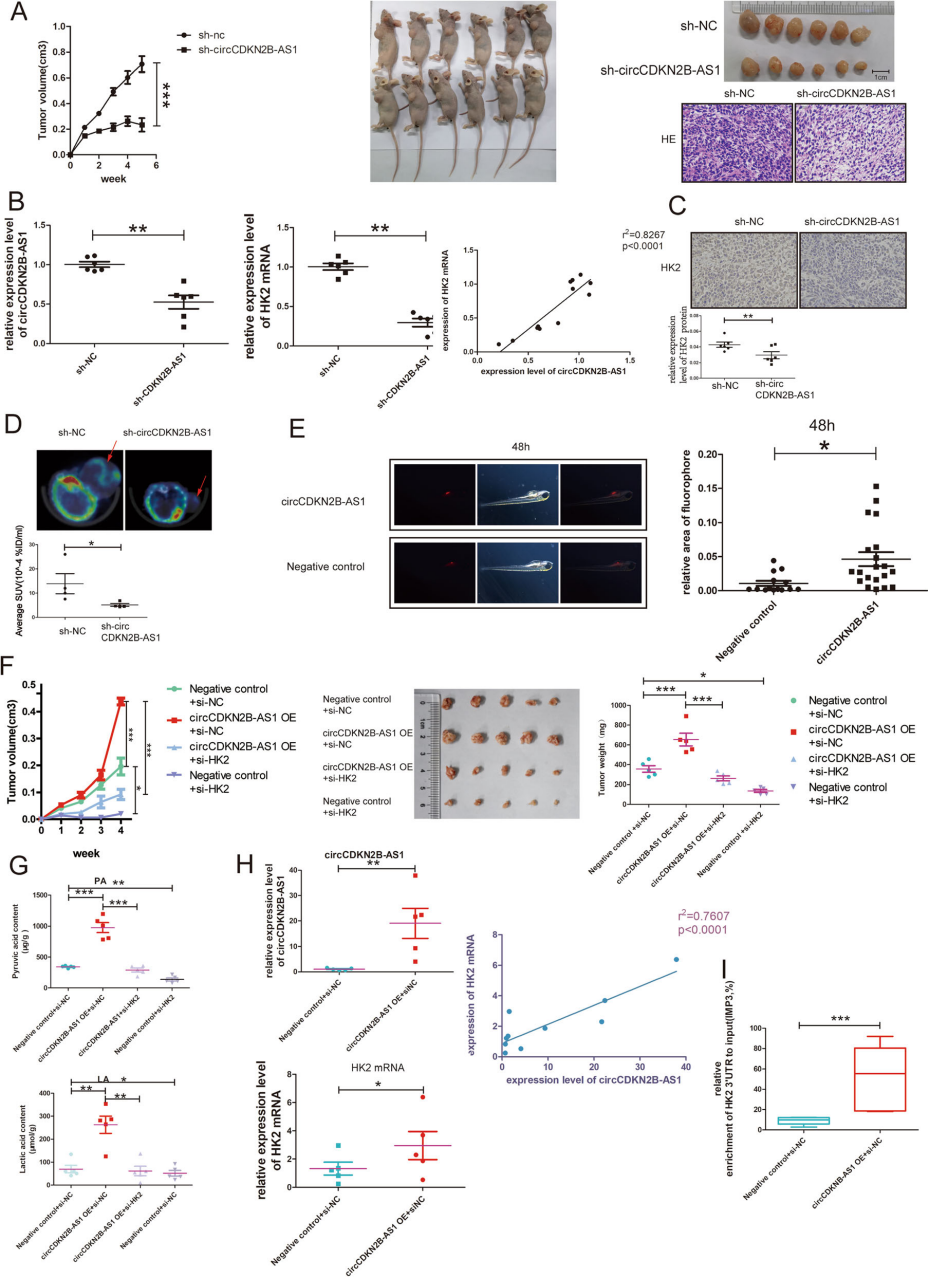
CircCDKN2B-AS1 regulates the expression of HK2 mRNA by recruiting IMP3 in vivo. **a** Left: the volume of subcutaneous tumors in nude mice with the sh-circCDKN2B-AS1 or negative control (mean ± SEM, *n* = 10, unpaired Student’s t-test); middle: images of mice sacrificed at week 5 after injection (n = 6); right upper: macro images of subcutaneous tumors; right lower: representative images of HE staining of subcutaneous tumors. **b** The levels of circCDKN2B-AS1(left) and HK2 mRNA (middle) expression in transplanted tumor tissues (mean ± SEM, n = 6, unpaired Student’s t-test). Right: statistical analyses of the association between circCDKN2B-AS1 and HK2 mRNA (*n* = 12, Pearson’s correlation coefficient). **c** Representative immunohistochemical staining images (upper) and quantification of HK2 protein expression (lower) in transplanted tumors (mean ± SEM, n = 6). **d** Upper: representative images of ^18^FDG-microPET/CT scans of transplanted tumors in nude mice. Lower: quantification of the average SUV of transplanted tumors (mean ± SEM, n = 4 each group, unpaired Student’s t-test). **e** Left: representative images of yolk sacs in zebrafish with circCDKN2B-AS1-overexpressing SiHa cells(*n* = 20) or negative controls (*n* = 14). Right: quantification of the relative area of fluorophores in zebrafish yolk sacs (unpaired Student’s t-test). **f** The volume of subcutaneous tumors in nude mice with negative control plus si-NC, circCDKN2B-AS1 overexpression plus si-NC, circCDKN2B-AS1 overexpression plus si-HK2, and negative control plus si-HK2 (mean ± SEM, *n* = 5, unpaired Student’s t-test). Macro images (middle) and weights (right) of transplanted tumors when mice were sacrificed at week 4 after tumor cell injection (n = 5, one-way ANOVA). **g** PA (upper) and LA (lower) contents of transplanted tumors (n = 5, one-way ANOVA, PA: μg/g, LA: μmol/g). **h** The levels of circCDKN2B-AS1 (left upper) and HK2 mRNA expression (left lower) in transplanted tumor tissues (mean ± SEM, n = 5, unpaired Student’s t-test). Right: the association between circCDKN2B-AS1 and HK2 mRNA (n = 10, Pearson’s correlation coefficient). **i** qRT-PCR following RIP assays showing the 3’UTR of HK2 mRNA recruited by the IMP3 protein in lysates of transplanted tumor tissues (min to max, n = 5, Kruskal-Wallis Test). *P < 0.05, **P < 0.01, ***P < 0.001

We constructed nude mouse models to observe circCDKN2B-AS1 effect on aerobic glycolysis and progression of cervical cancer, and found that the growth of xenografted tumors was markedly retarded (Fig. 6a) and the levels of both HK2 mRNA and protein expression were lower, with a significantly positive correlation between HK2 mRNA and circCDKN2B-AS1 expression (Fig. 6b-c), in 6 pairs of nude mice with depletion of circCDKN2B-AS1 compared to controls. Another 4 pairs of tumor-bearing nude mice were subjected to ^18^F-FDG microPET/CT imaging to assess the level of glucose metabolism in xenograft tumors. The average SUV in tumor tissues with circCDKN2B-AS1 knockdown was lower than that in control tissues (Fig. 6d). Then, we injected SiHa cells with upregulated circCDKN2B-AS1 into the yolk sac of zebrafish embryos and found an increased survival rate of cells with upregulated circCDKN2B-AS1 compared with control cells (Fig. 6e).

Further, we constructed additional 4 groups of nude mouse models with subcutaneous tumors that were circCDKN2B-AS1 overexpressed or not, and found that circCDKN2B-AS1 overexpression promoted tumor growth (Fig. 6f)), elevated LA and PA contents (Fig. 6g), elevated the level of HK2 mRNA expression of in tumor tissues with a positive correlation between them (Fig. 6h), and facilitated recruitment rate of HK2 mRNA 3’UTR to IMP3 (Fig. 6i, Additional file 11: Fig. S6). Then, siRNAs targeting NC or HK2 were injected into the subcutaneous tumor every week from day 7 after SiHa cell injection. We found that HK2 knockdown revised tumor growth and the LA/PA content promoted by circCDKN2B-AS1 overexpression (Fig. 6f, Fig. 6i). Thus, our results suggest that circCDKN2B-AS1 facilitates aerobic glycolysis and the progression of cervical cancer via interacting with IMP3 and HK2 mRNA in vivo.

## Discussion

CircRNAs are noncoding RNAs that are more stable than their linear isoforms. It has been reported that circRNAs are dysregulated and play a tumor suppressive or oncogenic role in cancers, such as breast cancer, ovarian cancer and bladder cancer [16, 43, 44]. Here, we identified a circRNA, circCDKN2B-AS1, that is upregulated in HPV16-positive cervical cancer and precancerous tissues. Furthermore, we showed that circCDKN2B-AS1 acts as a tumor promoter in cervical cancer development.

As circCDKN2B-AS1 is mainly localized in the cytoplasm of cervical cancer cells, we suspected that circCDKN2B-AS1 binds to microRNAs or RBPs, as most circRNAs do [44–48]. Then, we confirmed that IMP3 was the protein partner of circCDKN2B-AS1 by conducting a series of RNA-protein binding validation experiments. Our results indicated that HK2 was a common downstream molecule of circCDKN2B-AS1 and IMP3.

**Fig. 7:**
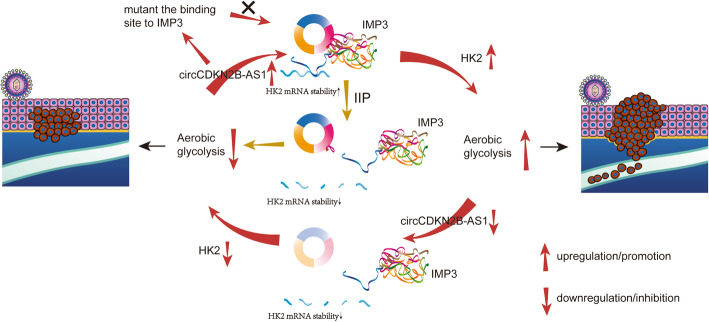
Schematic diagram for this article. The schematic diagram shows that adequate amounts of endogenous circCDKN2B-AS1 are produced with the development of cervical cancer. The IMP3 protein is recruited by circCDKN2B-AS1 to bind to the 3’UTR of HK2 mRNA, thereby stabilizing HK2 mRNA and promoting aerobic glycolysis and the malignant phenotype of cervical cancer. Knockdown of circCDKN2B-AS1 and mutation or blockade of the binding site between circCDKN2B-AS1 and IMP3 can impede the binding of IMP3 to the 3’UTR of HK2 mRNA, reduce HK2 expression levels and suppress aerobic glycolysis and progression in cervical cancer in vivo and in vitro

Thus, we hypothesized that circCDKN2B-AS1 stabilizes HK2 mRNA and facilitates aerobic glycolysis in cervical cancer by recruiting the IMP3 protein to the 3’UTR of HK2 mRNA (Fig. 7). Supporting this hypothesis, we showed that knockdown of circCDKN2B-AS1 weakened the binding of IMP3 to the 3’UTR of HK2 mRNA, reduced the stability of HK2 mRNA, decreased the expression of HK2 and suppressed aerobic glycolysis. In contrast, overexpression of circCDKN2B-AS1 had the opposite effects. However, overexpression of circCDKN2B-AS1 lacking the IMP3 binding site did not yield the above effects. Next, we showed that circCDKN2B-AS1 knockdown decreased the level of glucose metabolism and the expression of HK2 in xenograft tumors. Additionally, there was a significant positive correlation between the expression of circCDKN2B-AS1 and HK2 mRNA in xenografted tumors regardless circCDKN2B-AS1 knockdown or overexpression, suggesting that circCDKN2B-AS1 facilitates cellular malignant phenotypes of cervical cancer via interacting with IMP3 and HK2 mRNA in vivo.

Furthermore, we designed an inhibitory peptide, IIP, to block the interaction between circCDKN2B-AS1 and IMP3. We designed IIP based on the evidence that RRM1 of IMP3 is the key domain involved in binding CA-rich RNA elements [22, 23]. Our results showed that the inhibitory peptide IIP blocked the interaction between circCDKN2B-AS1 and IMP3, weakened the binding of IMP3 to the 3’UTR of HK2 mRNA, decreased the expression of HK2 and suppressed aerobic glycolysis.

There were some limitations of our study. For instance, it needs to be determined whether some of the potential circCDKN2B-AS1 binding proteins, such as IMP2, are involved in the gene regulatory network that promotes aerobic glycolysis in cervical cancer cells. In addition, the role of circCDKN2B-AS1 in other tumors and other diseases also needs to be investigated in the future.

## Conclusions

In the study, we found, for the first time to our knowledge, that circCDKN2B-AS1 acted as a promoter that facilitated aerobic glycolysis by sponging IMP3 protein to stabilize HK2 mRNA, consequently promoted malignant phenotypes in cervical cancer in vitro and in vivo, and the synthesizing inhibitory peptide IIP could effectively block the interaction between circCDKN2B-AS1 and IMP3 protein. Our findings may provide a new approach for cervical cancer therapeutics.

## Supplementary Information

**Additional file 1: Table S1.** Patient information for all specimens.

**Additional file 2: Table S2.** Sequences of primers, probes, siRNAs and peptides.

**Additional file 3: Fig. S1**. Identification of circCDKN2B-AS1, circFNACB, circEPSTI1 and circOBSL1. (A) Sequencing analysis of products amplified by the divergent primers showed the junction in circFNACB. (B) Sequencing analysis of products amplified by the divergent primers showed the junction in circEPSTI1. (C) Sequencing analysis of products amplified by the divergent primers showed the junction in circOBSL1. (D) Linear and circular isoforms of CDKN2B-AS1 were amplified from cDNA or gDNA from SiHa cells with the convergent primers and divergent primers, respectively. (E) The backsplicing point-specific probe was used in Northern blot analysis to detect endogenous circCDKN2B-AS1 in cervical cancer cell lines. Left lane: RNA molecular weight markers (2661, 1821, and 1517); right lane: circCDKN2B-AS1-specific probe.

**Additional file 4: Table S3.** Sanger sequencing results of PCR products.

**Additional file 5: Fig. S2.** CircCDKN2B-AS1 knockdown suppresses EMT of cervical cancer cells. (A) E-cadherin and β-Catenin protein expression levels in SiHa (left) and CaSki (right) cells were analyzed by Western blotting after transfection with two circCDKN2B-AS1 backsplicing-specific siRNAs or a negative control siRNA. GAPDH served as a loading control.

**Additional file 6: Fig. S3**. CircCDKN2B-AS1 overexpression facilitates the growth and vitality of cervical cancer cells. (A) The expression levels of circCDKN2B-AS1 in SiHa (left panel) and CaSki (right panel) cells with or without circCDKN2B-AS1 overexpression were determined by qRT-PCR (mean ± SEM, *n* = 4, unpaired Student’s t-test). (B) The growth curve of SiHa and CaSki cells transfected with circCDKN2B-AS1 overexpression plasmids or negative control plasmids was determined with CCK-8 assays. Upper panel: SiHa cells; lower panel: CaSki cells (mean ± SEM, *n* = 3, unpaired Student’s t-test). (C) The apoptosis level of SiHa and CaSki cells transfected with circCDKN2B-AS1 overexpression plasmids or negative control plasmids. Upper panel: SiHa cells; lower panel: CaSki cells. Right panel: quantification results of early apoptosis (mean ± SEM, *n* = 5, unpaired Student’s t-test). (D) Representative images (left panel) and quantification (right panel) of migration assays showing the migration capability of SiHa and CaSki cells transfected with circCDKN2B-AS1 overexpression plasmids or negative control plasmids (mean ± SEM, n = 5, unpaired Student’s t-test). (E) Representative images (left panel) and quantification (right panel) of Matrigel invasion assays showing the invasion capability of SiHa and CaSki cells transfected with circCDKN2B-AS1 overexpression plasmids or negative control plasmids (mean ± SEM, n = 5, unpaired Student’s t-test). *P < 0.05, **P < 0.01, ***P < 0.001.

**Additional file 7: Fig. S4**. CircCDKN2B-AS1 cooperates with IMP3 to promote glycolysis in cervical cancer. (A) Analysis of differential bands in Fig. 3b. Left upper: the partial magnification of the glue drawing in Fig. 3b.The red arrow point marks the band we chose (Band1),and the blue marks the other two increased bands (Band2 and Band3); Left lower: the Gray value distribution map analyzed by using image J software; Right: quantification of the ratio of grey value between the two groups in the differential bands. (B) Silver staining indicating the differential proteins pulled down by the circCDKN2B-AS1 junction-specific probe and the oligo probe in CaSki cells. The red box indicating the differential proteins. (C) Western blot assay showing that the IMP3 protein was pulled down by biotin-labeled circCDKN2B-AS1 probes from the lysates of CaSki cells. (D) The ECAR was measured in SiHa (left upper) and CaSki (left lower) cells transfected with two junction-specific siRNAs or a negative control siRNA with Seahorse XFe assays. Right panel: quantification of glycolytic capacity and glycolysis in SiHa (right upper) and CaSki (right lower) cells (mean ± SEM, *n* = 6, one-way ANOVA). (E) Quantification of the %PER from the glycolysis of SiHa (left panel) and CaSki (right panel) cells transfected with negative control plasmids, circCDKN2B-AS1-overexpressing plasmids and mutant circCDKN2B-AS1-overexpressing plasmids (mean ± SEM, n = 6, one-way ANOVA). (F) The ECAR was measured in SiHa (left upper) and CaSki (left lower) cells transfected with the circCDKN2B-AS1 overexpression vector or the negative control vector with Seahorse XFe assays. Right panel: quantification of glycolytic capacity and glycolysis in SiHa (right upper) and CaSki (right lower) cells (mean ± SEM, n = 6, unpaired Student’s t-test). *P < 0.05, **P < 0.01, ***P < 0.001.

**Additional file 8: Table S4.** All proteins verified by mass spectrometry analysis.

**Additional file 9: Table S5.** All proteins predicted by RBPmap.

**Additional file 10: Fig. S5**. CircCDKN2B-AS1 regulates the stability of HK2 mRNA by recruiting IMP3. (A) The expression levels of HK2, ENO1, PGK1 and PKM in SiHa cells with or without circCDKN2B-AS1 knockdown were determined by qRT-PCR (mean ± SEM, n = 5, one-way ANOVA). (B) HK2 mRNA (lower) and HK2 protein (upper) levels in SiHa cells stably transfected with sh-circCDKN2B-AS1 or negative control and HK2 overexpression plasmids (mean ± SEM, n = 3, unpaired Student’s t-test). *P < 0.05, **P < 0.01, ***P < 0.001.

**Additional file 11: Fig. S6.** The expression levels of circCDKN2B-AS1 and IMP3 protein in in transplanted tumor tissues. The levels of IMP3 protein expression (left) in transplanted tumor tissues (mean ± SEM, n = 6, unpaired Student’s t-test). Right: the association between circCDKN2B-AS1 and IMP3 protein expressions (*n* = 12, Pearson’s correlation coefficient).

## Data Availability

The datasets supporting the conclusions of this article are included within the article (and its additional files).

## Abbreviations

ATCC: American type culture collection
CircRNAs: Circular RNAs
CDKN2B-AS1: CDKN2B antisense
RNA 1: CircCDKN2B-AS1:Circular isoform of CDKN2B antisense RNA 1
HPV: Human papillomavirus
IMP3/IGF2BP3: Insulin like growth factor 2 mRNA binding protein 3
HK2: Hexokinase 2
ENO1: Enolase 1
PKG1: Phosphoglycerate kinase 1
PKM: Pyruvate kinase M 1/2
qRT-PCR: Quantitative real-time polymerase chain reaction
3′-UTR: 3′ end Untranslated Regions
FCM: Flow cytometry analysis
CCK8: Cell counting kit-8
MS: Mass spectrometry
RNA-Seq: Genome-wide transcriptome analysis
FITC: Fluorescein isothiocyanate
GAPDH: Glyceraldehyde-3-phosphate dehydrogenase
RIP: RNA Binding Protein Immunoprecipitation
PET: Positron emission tomography
CT: Computed Tomography
RNA FISH: RNA Fluorescence in situ hybridization
mRNA: Message RNA
CLIP-seq: Crosslinking-immunprecipitation and high-throughput sequencing
NC: Negative control
IIP: Inhibitory IMP3 peptide
CTL: Control peptide
shRNA: Short hairpin RNA
siRNA: Small interference RNA
cDNA: Complementary DNA
gDNA: Genomic DNA
LA: Lactic acid
PA: Pyruvic acid
EMT: Epithelial-mesenchymal transition
