# Supplementary figures and images for: CircCDKN2B-AS1 interacts with IMP3 to stabilize hexokinase 2 mRNA and facilitate cervical squamous cell carcinoma aerobic glycolysis progression
Source: J Exp Clin Cancer Res. 2020 Dec 11;39:281. doi: 10.1186/s13046-020-01793-7 (PMC7731507; doi:10.1186/s13046-020-01793-7)

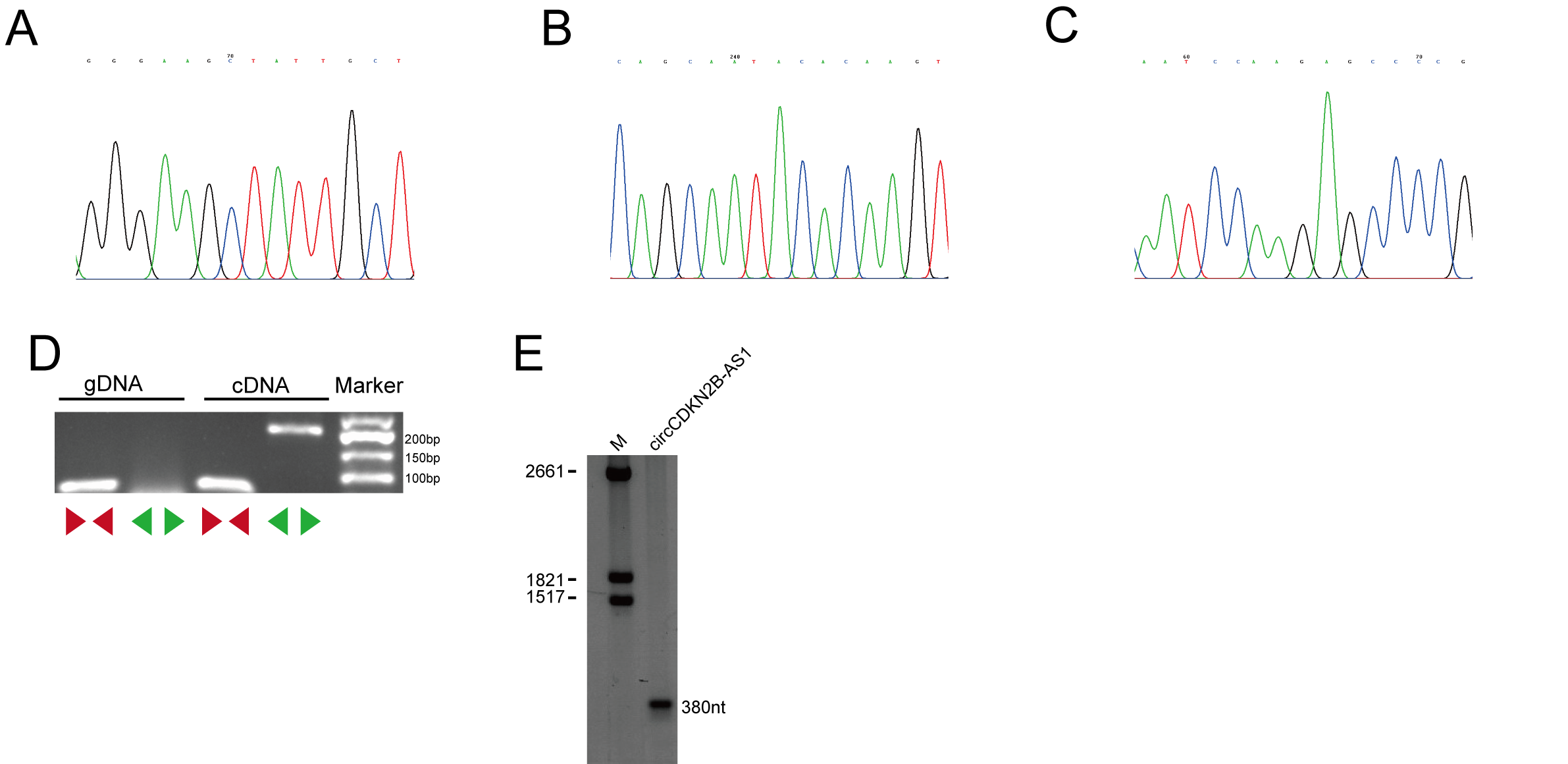

Supplement: Supplementary file 3 — Additional file 3: Fig. S1. Identification of circCDKN2B-AS1, circFNACB, circEPSTI1 and circOBSL1. (A) Sequencing analysis of products amplified by the divergent primers showed the junction in circFNACB. (B) Sequencing analysis of products amplified by the divergent primers showed the junction in circEPSTI1. (C) Sequencing analysis of products amplified by the divergent primers showed the junction in circOBSL1. (D) Linear and circular isoforms of CDKN2B-AS1 were amplified from cDNA or gDNA from SiHa cells with the convergent primers and divergent primers, respectively. (E) The backsplicing point-specific probe was used in Northern blot analysis to detect endogenous circCDKN2B-AS1 in cervical cancer cell lines. Left lane: RNA molecular weight markers (2661, 1821, and 1517); right lane: circCDKN2B-AS1-specific probe. [file 13046_2020_1793_MOESM3_ESM.tif]

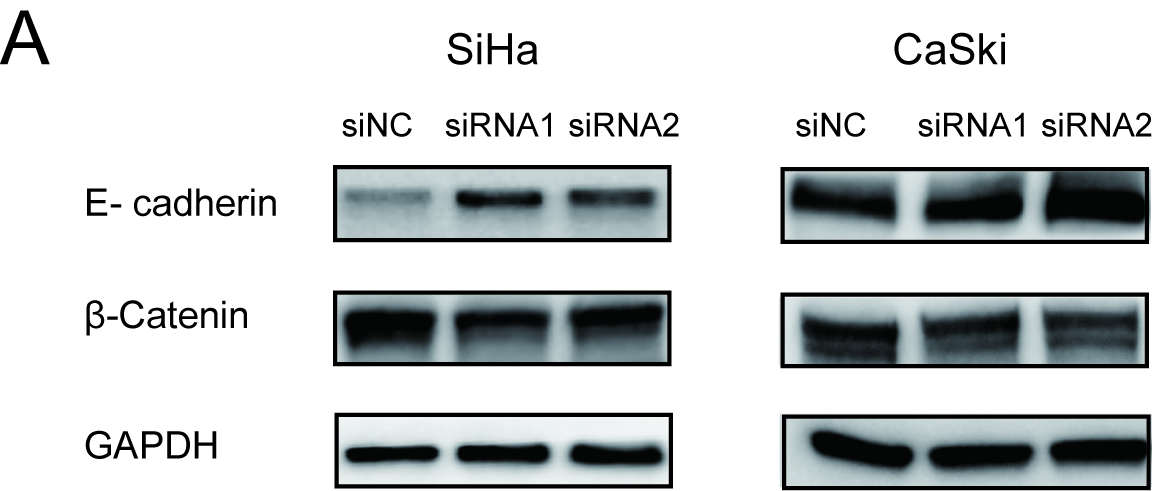

Supplement: Supplementary file 5 — Additional file 5: Fig. S2. CircCDKN2B-AS1 knockdown suppresses EMT of cervical cancer cells. (A) E-cadherin and β-Catenin protein expression levels in SiHa (left) and CaSki (right) cells were analyzed by Western blotting after transfection with two circCDKN2B-AS1 backsplicing-specific siRNAs or a negative control siRNA. GAPDH served as a loading control. [file 13046_2020_1793_MOESM5_ESM.tif]

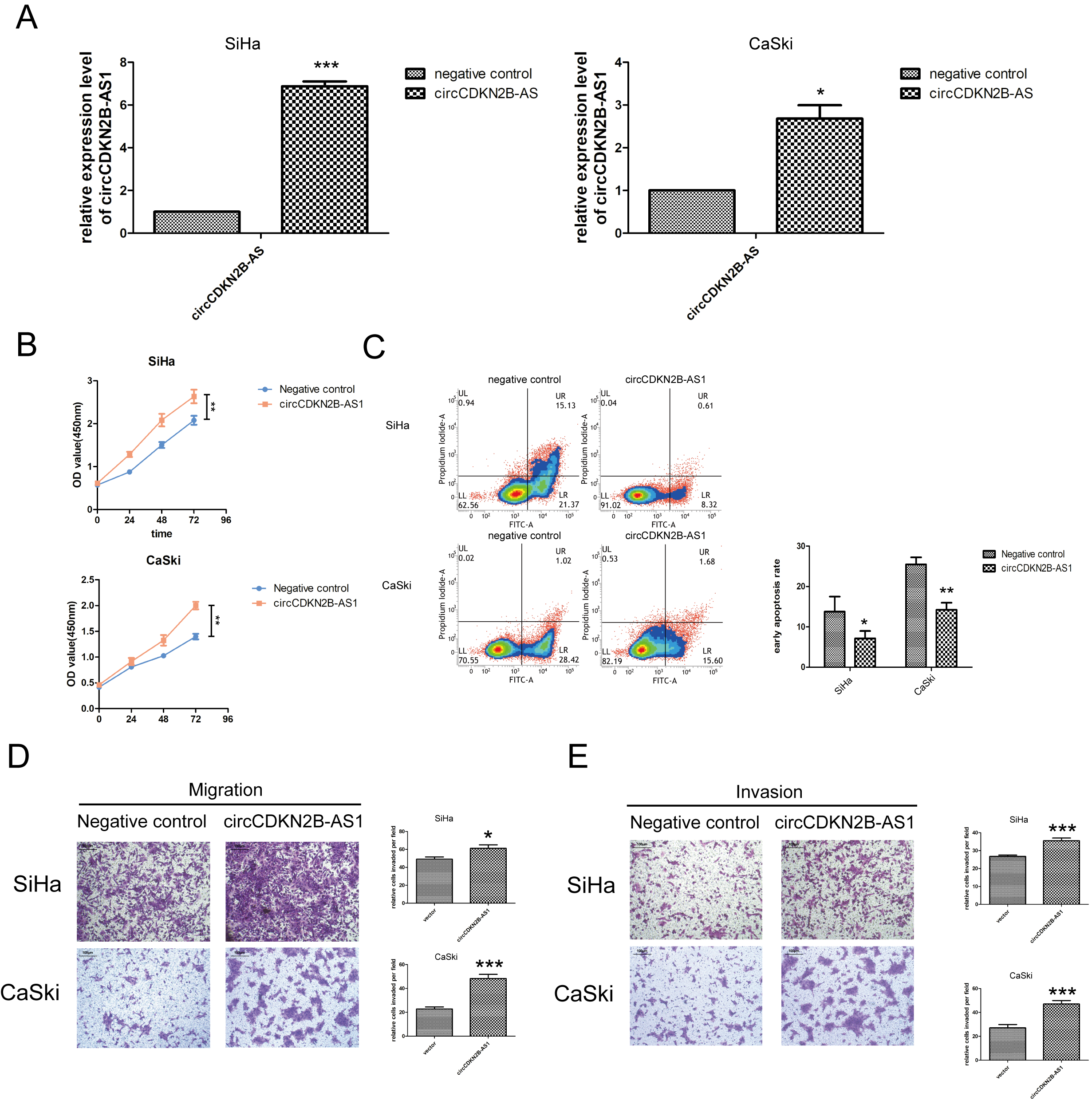

Supplement: Supplementary file 6 — Additional file 6: Fig. S3. CircCDKN2B-AS1 overexpression facilitates the growth and vitality of cervical cancer cells. (A) The expression levels of circCDKN2B-AS1 in SiHa (left panel) and CaSki (right panel) cells with or without circCDKN2B-AS1 overexpression were determined by qRT-PCR (mean ± SEM, n = 4, unpaired Student’s t-test). (B) The growth curve of SiHa and CaSki cells transfected with circCDKN2B-AS1 overexpression plasmids or negative control plasmids was determined with CCK-8 assays. Upper panel: SiHa cells; lower panel: CaSki cells (mean ± SEM, n = 3, unpaired Student’s t-test). (C) The apoptosis level of SiHa and CaSki cells transfected with circCDKN2B-AS1 overexpression plasmids or negative control plasmids. Upper panel: SiHa cells; lower panel: CaSki cells. Right panel: quantification results of early apoptosis (mean ± SEM, n = 5, unpaired Student’s t-test). (D) Representative images (left panel) and quantification (right panel) of migration assays showing the migration capability of SiHa and CaSki cells transfected with circCDKN2B-AS1 overexpression plasmids or negative control plasmids (mean ± SEM, n = 5, unpaired Student’s t-test). (E) Representative images (left panel) and quantification (right panel) of Matrigel invasion assays showing the invasion capability of SiHa and CaSki cells transfected with circCDKN2B-AS1 overexpression plasmids or negative control plasmids (mean ± SEM, n = 5, unpaired Student’s t-test). *P < 0.05, **P < 0.01, ***P < 0.001. [file 13046_2020_1793_MOESM6_ESM.tif]

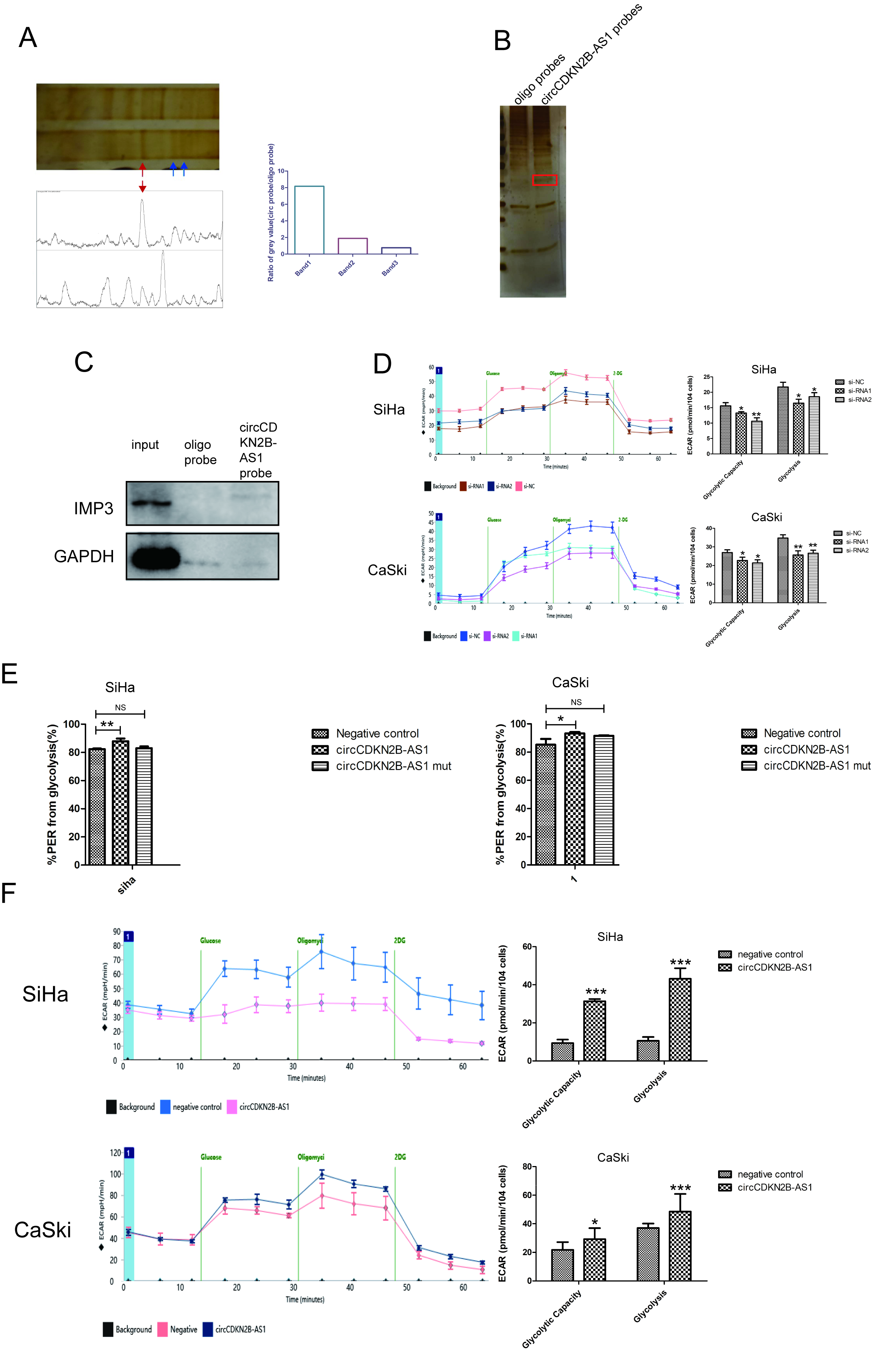

Supplement: Supplementary file 7 — Additional file 7: Fig. S4. CircCDKN2B-AS1 cooperates with IMP3 to promote glycolysis in cervical cancer. (A) Analysis of differential bands in Fig. 3b. Left upper: the partial magnification of the glue drawing in Fig. 3b.The red arrow point marks the band we chose (Band1),and the blue marks the other two increased bands (Band2 and Band3); Left lower: the Gray value distribution map analyzed by using image J software; Right: quantification of the ratio of grey value between the two groups in the differential bands. (B) Silver staining indicating the differential proteins pulled down by the circCDKN2B-AS1 junction-specific probe and the oligo probe in CaSki cells. The red box indicating the differential proteins. (C) Western blot assay showing that the IMP3 protein was pulled down by biotin-labeled circCDKN2B-AS1 probes from the lysates of CaSki cells. (D) The ECAR was measured in SiHa (left upper) and CaSki (left lower) cells transfected with two junction-specific siRNAs or a negative control siRNA with Seahorse XFe assays. Right panel: quantification of glycolytic capacity and glycolysis in SiHa (right upper) and CaSki (right lower) cells (mean ± SEM, n = 6, one-way ANOVA). (E) Quantification of the %PER from the glycolysis of SiHa (left panel) and CaSki (right panel) cells transfected with negative control plasmids, circCDKN2B-AS1-overexpressing plasmids and mutant circCDKN2B-AS1-overexpressing plasmids (mean ± SEM, n = 6, one-way ANOVA). (F) The ECAR was measured in SiHa (left upper) and CaSki (left lower) cells transfected with the circCDKN2B-AS1 overexpression vector or the negative control vector with Seahorse XFe assays. Right panel: quantification of glycolytic capacity and glycolysis in SiHa (right upper) and CaSki (right lower) cells (mean ± SEM, n = 6, unpaired Student’s t-test). *P < 0.05, **P < 0.01, ***P < 0.001. [file 13046_2020_1793_MOESM7_ESM.tif]

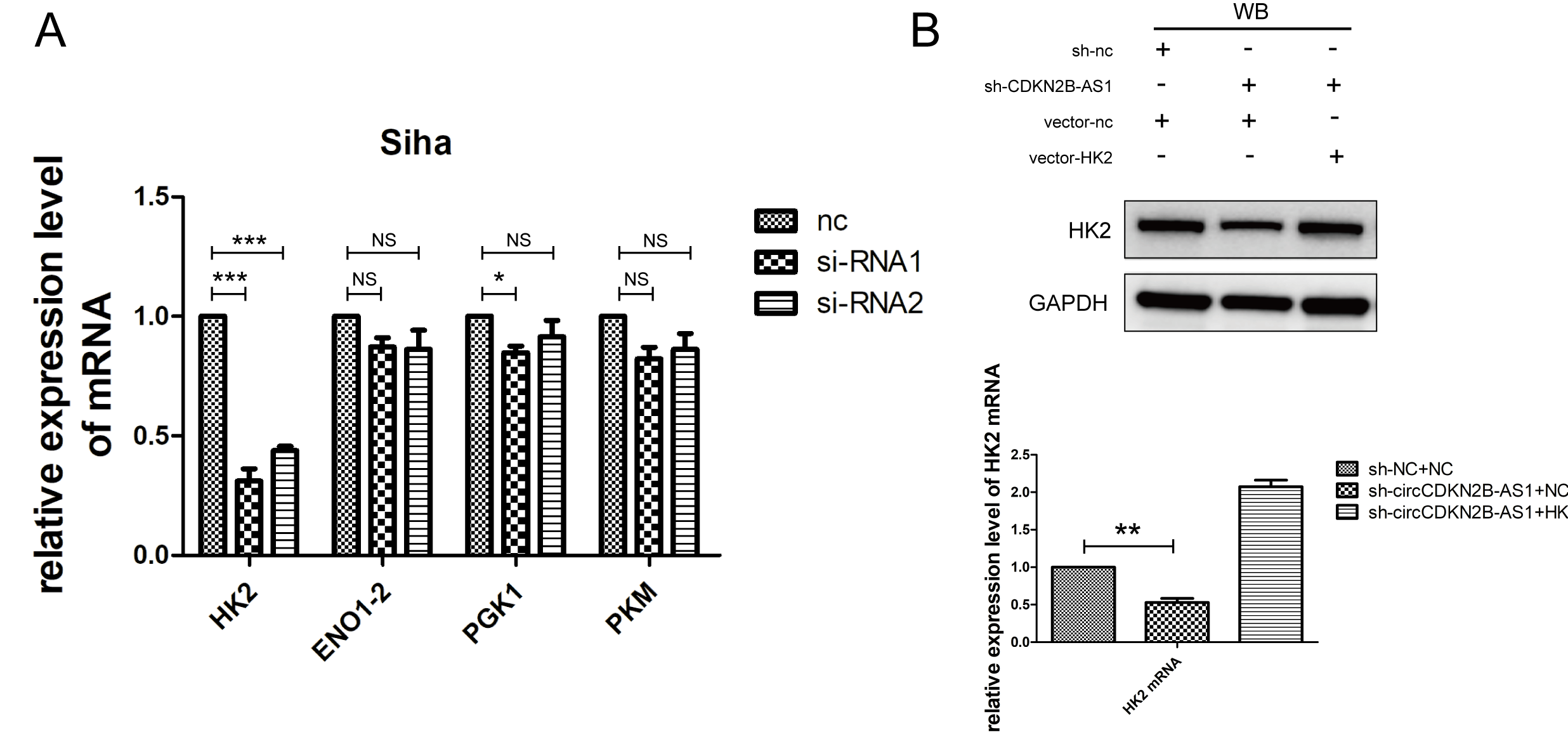

Supplement: Supplementary file 10 — Additional file 10: Fig. S5. CircCDKN2B-AS1 regulates the stability of HK2 mRNA by recruiting IMP3. (A) The expression levels of HK2, ENO1, PGK1 and PKM in SiHa cells with or without circCDKN2B-AS1 knockdown were determined by qRT-PCR (mean ± SEM, n = 5, one-way ANOVA). (B) HK2 mRNA (lower) and HK2 protein (upper) levels in SiHa cells stably transfected with sh-circCDKN2B-AS1 or negative control and HK2 overexpression plasmids (mean ± SEM, n = 3, unpaired Student’s t-test). *P < 0.05, **P < 0.01, ***P < 0.001. [file 13046_2020_1793_MOESM10_ESM.tif]

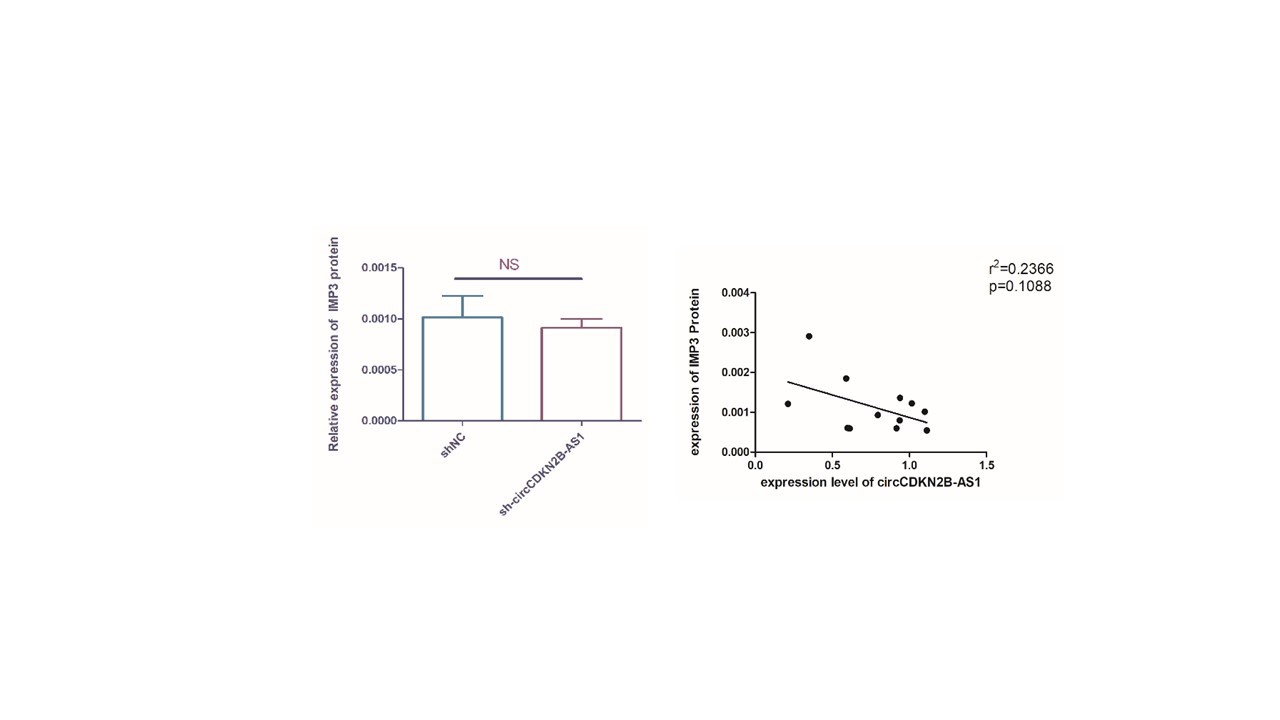

Supplement: Supplementary file 11 — Additional file 11: Fig. S6. The expression levels of circCDKN2B-AS1 and IMP3 protein in in transplanted tumor tissues. The levels of IMP3 protein expression (left) in transplanted tumor tissues (mean ± SEM, n = 6, unpaired Student’s t-test). Right: the association between circCDKN2B-AS1 and IMP3 protein expressions (n = 12, Pearson’s correlation coefficient). [file 13046_2020_1793_MOESM11_ESM.jpg]
