# Supplementary material for: CircCDKN2B-AS1 interacts with IMP3 to stabilize hexokinase 2 mRNA and facilitate cervical squamous cell carcinoma aerobic glycolysis progression
Source: J Exp Clin Cancer Res. 2020 Dec 11;39:281. doi: 10.1186/s13046-020-01793-7 (PMC7731507; doi:10.1186/s13046-020-01793-7)
Supplement: Supplementary file 4 — Additional file 4: Table S3. Sanger sequencing results of PCR products. [file 13046_2020_1793_MOESM4_ESM.docx]

**circRNA12542 circCDKN2B-AS1 chr9:22046750-22097363**

**2、3、4 exons of CDKN2B-AS1 transtripts variants 4**

TGTCCCTTTTGATGAGAAGAATAAGCCTCATTCTGATTCAACAGCAGAGATCAAAGAAAAGACTTCTGTTTTCTGGCCACCAGATATATGTTATCTGTGCTTAAAGAATTGAAAAACACACATCAAAGGAGAATTTTCTTGGAAAGAGAGGGTTCAAGCATCACTGTTAGGTGTGCTGGAATCCTTTCCCGAGTCAGTACTGCTTTCTAGAAGAAAACCGGGGAGATCTATTTGGAATGTATCTAACTCCAAAGAAACCATCAGAGGTAACAGGTGGAGAACTTCAGTAGAGGAAGTGGCAGGAATTTGGGAATGAGGAGCACAGTGATTAAACTGGGGCCATTCATATGAGAGTTTAAGAACTCAGACCAGTGACTTAG

**Sanger sequencing of products amplified by divergent primers**

T6-1_M13F_TSS20170915-0571-1747_A07

GCGACGTCTGAGCTTGATATCGAATTCGCGTGTCGCCCTTTCAGTAGAGGAAGTGGCAGGAATTTGGGAATGAGGAGCACAGTGATTAAACTGGGGCCATTCATATGAGAGTTTAAGAACTCAGACCAGTGACTTAGTGTCCCTTTTGATGAGAAGAATAAGCCTCATTCTGATTCAACAGCAGAGATCAAAGAAAAGACTTCTGTTTTCTGGCCACCAGATATATGTTATCTGTGCTTAAAGAATTGAAAAACACACATCAAAGGAGAATTTTCTTGGAAAGAGAGGGTTCAAGCATCAAAGGGCGACACGCGAATTCGATATCGCGGCCGCCTGCAGTCAATACTGACGATGGTCATAGCTGTTTCCTGTCCATAGCAGAAAGTCAAAAGCCTCCGACCGGAGGCTTTTGACTTGATCGGCACGTAAGAGGTTCCAACTTTCACCATAATGAAATAAGATCACTACCGGGCGTATTTTTTGAGTTATCGAGATTTTCAGGAGCTAAGGAAGCTAAAATGAGTATTCAACATTTCCGTGTCGCACTTATTCCGTTTTTTGCGGCATTTTGCCTTCCTGTTTTTGCTCACCCAGAAACGCTGGTGAAAGTAAAAGATGCTGAAGATCAGTTGGGTGCACGAGTGGGTTACATCGAACTGGATCTCAACAGCGGTAAGATCCTTGAGAGTTTTCGCCCCGAAGAACGTTTTCCAATGATGAGCACTTTTAAAGTTCTGCTATGTGGCGCGGTATTATCCCGTATTGACGCCGGGCAAGAGCAACTCGGTCGCCGCATACACTATTCTCAGAATGACTTGGTTGAGTACTCACCAGTCACAGAAAAGCATCTTACGGATGGCATGACAGTAAGAGAATTATGCAGTGCTGCCATAACCATGAGTGATAACACTGCGGCCAACTTACTTCTGACAACGATCGGAGGACCGAAGGAGCTAACCGCTTTTTTGCCCACATGGGGGATC

T6-2_M13R_TSS20170915-0571-1747_G08

GATGCGGACGCGATATCGAATTCGCGTGTCGCCCTTTCAGTAGAGGAAGTGGCAGGAATTTGGGAATGAGGAGCACAGTGATTAAACTGGGGCCATTCATATGAGAGTTTAAGAACTCAGACCAGTGACTTAGTGTCCCTTTTGATGAGAAGAATAAGCCTCATTCTGATTCAACAGCAGAGATCAAAGAAAAGACTTCTGTTTTCTGGCCACCAGATATATGTTATCTGTGCTTAAAGAATTGAAAAACACATATCAAAGGAGAATTTTCTTGGAAAGAGAGGGTTCAAGCATCAAAGGGCGACACGCGAATTCGATATCAAGCTTCAGGACTGCAGCGAGCCTCAGACACTGGCCGTCGTTTTACACAATCAACTCACTGGCTCACCTTCACGGGTGGGCCTTTCTTCGGTAGAAAATCAAAGGATCTTCTTGAGATCCTTTTTTTCTGCGCGTAATCTGCTGCTTGCAAACAAAAAAACCACCGCTACCAGCGGTGGTTTGTTTGCCGGATCAAGAGCTACCAACTCTTTTTCCGAGGTAACTGGCTTCAGCAGAGCGCAGATACCAAATACTGTTCTTCTAGTGTAGCCGTAGTTAGGCCACCACTTCAAGAACTCTGTAGCACCGCCTACATACCTCGCTCTGCTAATCCTGTTACCAGTGGCTGCTGCCAGTGGCGATAAGTCGTGTCTTACCGGGTTGGACTCAAGACGATAGTTACCGGATAAGGCGCAGCGGTCGGGCTGAACGGGGGGTTCGTGCACACAGCCCAGCTTGGAGCGAACGACCTACACCGAACTGAGATACCTACAGCGTGAGCTATGAGAAAGCGCCACGCTTCCCGAAGGGAGAAAGGCGGACAGGTATCCGGTAAGCGGCAGGGTCGGAACAGGAGAGCGCACGAGGGAGCTTCCAGGGGGAAACGCCTGGTATCTTTATAGTCCTGTCGGGTTTCGCCACCTCTGACTTGAACATCGATTTTTGGGATGCTCGTCAGGGGGGCGGAACCCATGGAAAAA

**Sanger sequencing of products amplified by full-length primers**

>TW5_TSS20171130-0571-3655.seq.Contig1

GTAGAGGAAGTGGCAGGAATTTGGGAATGAGGAGCACAGTGATTAAACTG

GGGCCATTCATATGAGAGTTTAAGAACTCAGACCAGTGACTTAGTGTCCC

TTTTGATGAGAAGAATAAGCCTCATTCTGATTCAACAGCAGAGATCAAAG

AAAAGACTTCTGTTTTCTGGCCACCAGATATATGTTATCTGTGCTTAAAG

AATTGAAAAACACACATCAAAGGAGAATTTTCTTGGAAAGAGAGGGTTCA

AGCATCACTGTTAGGTGTGGTGGAATCCTTTCCCGAGTCAGTACTGCTTT

CTAGAAGAAAACCGGGGAGATCTATTTGGAATGTATCTAACTCCAAAGAA

A

>TW7_TSS20171130-0571-3655.seq.Contig1

TTCAGTAGAGGAAGTGGCAGGAATTTGGGAATGAGGAGCACAGTGATTAA

ACTGGGGCCATTCATATGAGAGTTTAAGAACTCAGACCAGTGACTTAGTG

TCCCTTTTGATGAGAAGAATAAGCCTCATTCTGATTCAACAGCAGAGATC

AAAGAAAAGACTTCTGTTTTCTGGCCACCAGATATATGTTATCTGTGCTT

AAAGAATTGAAAAACACACATCAAAGGAGAATTTTCTTGGAAAGAGAGGG

TTCAAGCATCACTGTTAGGTGTGGTGGAATCCTTTCCCGAGTCAGTACTG

CTTTCTAGAAGAAAACCGGGGAGATCTATTTGGAATGTATCTAACTCCAA

AGA

**Sanger sequencing of products amplified by divergent primers after overexpression of circCDKN2B-AS1**

B04_S28214_S291209a_C-2_M13F(-47)

TTGGGAACGGGCCAGTGAATTCGAGCTCGGTACCCGGGGATCCTCTAGAGATTTCAGTAGAGGAAGTGGCAGGAATTTGG

GAATGAGGAGCACAGTGATTAAACTGGGGCCATTCATATGAGAGTTTAAGAACTCAGACCAGTGACTTAGTGTCCCTTTT

GATGAGAAGAATAAGCCTCATTCTGATTCAACAGCAGAGATCAAAGAAAAGACTTCTGTTTTCTGGCCACCAGATATATG

TTATCTGTGCTTAAAGAATTGAAAAACACACATCAAAGGAGAATTTTCTTGGAAAGAGAGGGTTCAAGCATCAAATCGTC

GACCTGCAGGCATGCAAGCTTGGCGTAATCATGGTCATAGCTGTTTCCTGTGTGAAATTGTTATCCGCTCACAATTCCAC

ACAACATACGAGCCGGAAGCATAAAGTGTAAAGCCTGGGGTGCCTAATGAGTGAGCTAACTCACATTAATTGCGTTGCGC

TCACTGCCCGCTTTCCAGTCGGGAAACCTGTCGTGCCAGCTGCATTAATGAATCGGCCAACGCGCGGGGAGAGGCGGTTT

GCGTATTGGGCGCTCTTCCGCTTCCTCGCTCACTGACTCGCTGCGCTCGGTCGTTCGGCTGCGGCGAGCGGTATCAGCTC

ACTCAAAGGCGGTAATACGGTTATCCACAGAATCAGGGGATAACGCAgGAAAGAACATGTGAGCAAAAGGCCAGCAAAAG

GCCAGGAACCGTAAAAAGGCCGCGTTGCTGGCGTTTTTCCATAgGCTCCGCCCCCCTGACGAGCATCACAAAAATCGACG

CTCAAGTCAGAGGTGGCGAAACCCGACAGGACTATAAAGATACCAGGCGTTTCCCCCTGGAAGCTCCCTCGTGCGCTCTC

CTGTTCCGACCCTGCCGCTTACCGGATACCTGTCCGCCTTTCTCCCTTCGGGAGCGTGGCGCTTTCTCATAGCTCACGCT

GTAGGTATCTCAGTTCGGTGTAGTCGTTCGCTCCAAGCTGGGCTGTGTGCACGAACCCCCCGTTCAGCCCGACGCTGCGC

CTTATC
